# Exploring Computational Data Amplification and Imputation for the Discovery of Type 1 Diabetes (T1D) Biomarkers from Limited Human Datasets

**DOI:** 10.3390/biom12101444

**Published:** 2022-10-09

**Authors:** Oscar Alcazar, Mitsunori Ogihara, Gang Ren, Peter Buchwald, Midhat H. Abdulreda

**Affiliations:** 1 Diabetes Research Institute, University of Miami Miller School of Medicine, Miami, FL 33136, USA; 2 Institute for Data Science and Computing, University of Miami, Coral Gables, FL 33146, USA; 3 Department of Computer Science, University of Miami, Coral Gables, FL 33146, USA; 4 Department of Molecular and Cellular Pharmacology, University of Miami Miller School of Medicine, Miami, FL 33136, USA; 5 Department of Surgery, University of Miami Miller School of Medicine, Miami, FL 33136, USA; 6 Department of Microbiology and Immunology, University of Miami Miller School of Medicine, Miami, FL 33136, USA; 7 Department of Ophthalmology, University of Miami Miller School of Medicine, Miami, FL 33136, USA

**Keywords:** artificial intelligence (AI), algorithm, big data, data imputation and amplification, early biomarker signatures, early diagnosis, integrated analysis, lipidomics, multi-omics, metabolomics, machine learning (ML), prevention, proteomics, transcriptomics, type 1 diabetes (T1D)

## Abstract

Background: Type 1 diabetes (T1D) is a devastating disease with serious health complications. Early T1D biomarkers that could enable timely detection and prevention before the onset of clinical symptoms are paramount but currently unavailable. Despite their promise, omics approaches have so far failed to deliver such biomarkers, likely due to the fragmented nature of information obtained through the single omics approach. We recently demonstrated the utility of parallel multi-omics for the identification of T1D biomarker signatures. Our studies also identified challenges. Methods: Here, we evaluated a novel computational approach of data imputation and amplification as one way to overcome challenges associated with the relatively small number of subjects in these studies. Results: Using proprietary algorithms, we amplified our quadra-omics (proteomics, metabolomics, lipidomics, and transcriptomics) dataset from nine subjects a thousand-fold and analyzed the data using *Ingenuity Pathway Analysis (IPA)* software to assess the change in its analytical capabilities and biomarker prediction power in the amplified datasets compared to the original. These studies showed the ability to identify an increased number of T1D-relevant pathways and biomarkers in such computationally amplified datasets, especially, at imputation ratios close to the “golden ratio” of 38.2%:61.8%. Specifically, the *Canonical Pathway* and *Diseases and Functions* modules identified higher numbers of inflammatory pathways and functions relevant to autoimmune T1D, including novel ones not identified in the original data. The *Biomarker Prediction* module also predicted in the amplified data several unique biomarker candidates with direct links to T1D pathogenesis. Conclusions: These preliminary findings indicate that such large-scale data imputation and amplification approaches are useful in facilitating the discovery of candidate integrated biomarker signatures of T1D or other diseases by increasing the predictive range of existing data mining tools, especially when the size of the input data is inherently limited.

## 1. Introduction

Type 1 diabetes (T1D) results from loss of the insulin-producing beta cells in the endocrine pancreas by a process referred to as autoimmunity. Autoimmunity is defined as an immune attack against one’s own organs/tissues. In T1D, the anti-beta cell autoimmunity typically begins early in life and, consequently, ~60% of T1D diagnoses are often rendered in children and young adults [1,2,3]. Since children are considered a vulnerable patient population and because T1D was considered for a long time as primarily a children’s disease, development of preventive therapies has been limited, and treatment implementation in at-risk children is approached very cautiously or avoided altogether until clinical diagnosis is confirmed. Unfortunately, any therapy initiated at clinical diagnosis is already at a significant disadvantage because substantial damage to the beta cell mass will have already occurred (i.e., “point-of-no-return”). Crossing this threshold leads to insulin insufficiency, loss of glucose homeostasis (dysglycemia), and increased blood sugar levels (hyperglycemia). Chronic dysglycemia and hyperglycemia also lead to serious health complications such as blindness, kidney failure, nerve damage, limb amputation, and even death. Despite improved management and control of diabetes, there is still excess mortality and loss of 10–20 life-years among those diagnosed with T1D [4,5], and all-cause mortality risk is about three-fold higher in them compared to the general population [6]; it is also more than four times in those who develop T1D before 10 years of age, where it is also further estimated to result in a loss of 17.7 life-years in women and 14.2 in men [4]. Therefore, early T1D biomarkers are crucial because they provide clarity on the cost–benefit calculation of whether to initiate (or not) powerful therapies before reaching the point-of-no-return. This is particularly important when such therapies could have serious risks and side-effects associated with them (e.g., immune modulating therapies). To date, however, there are no such early T1D biomarkers that can adequately discriminate among at-risk individuals who will or will not progress to clinical diagnosis. 

With recent advancements in omics approaches made possible by the rapid progress of quantitative analytics, the T1D research community has been actively searching for T1D biomarkers to complement known immunological ones, such as autoantibodies, through the application of various omics approaches such as genomics, proteomics, metabolomics, lipidomics, and transcriptomics [7,8]. The central idea behind using omics is that they can identify at-risk subjects based on specific genetic and biochemical disturbances that could serve as T1D biomarkers. Most of these studies have been conducted using single omics approaches and thus, unfortunately, have not produced biomarkers that can definitively inform decisions on early intervention. However, there is emerging recognition that this failure in identifying reliable early T1D biomarkers is likely due to the fragmented information on the complex T1D pathogenic processes obtained through single omics. Therefore, more recent efforts have been exploring the utility of combining information from two or more omics-type analyses [9,10]. We recently demonstrated the feasibility of the novel approach of parallel multi-omics and showed its potential in identifying candidate composite T1D biomarker signatures composed of combinations of proteins, metabolites, lipids, and gene transcripts identified as differentially affected in at-risk subjects in the integrated multi-omics analyses [11]. We performed simultaneous proteomics, metabolomics, lipidomics, and transcriptomics on the same blood samples (i.e., in parallel) from children at high risk of developing T1D and from healthy controls for comparison. The basic premise is that parallel multi-omics measurements can (1) provide a more comprehensive and consistent picture of the disturbances in at-risk subjects and, thus, (2) facilitate the identification of associated biomarker signatures. Moreover, if performed longitudinally during T1D progression, multi-omics may also identify stage-specific signatures of T1D pathogenesis, thereby providing further guidance on more-targeted treatment options in a timely fashion. 

**Figure 1:**
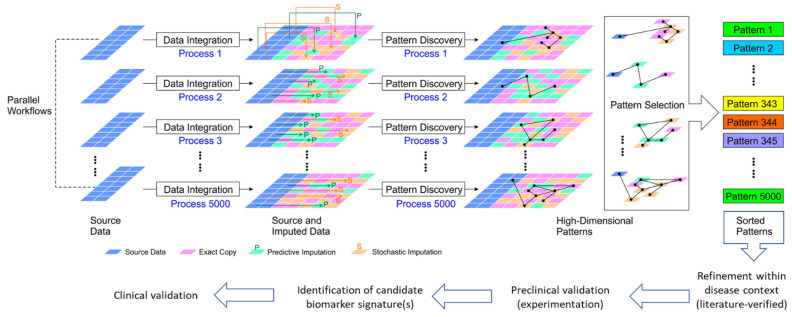
Schematic illustration of our proposed very-large-scale predictive data integration and imputation process for high-dimensional pattern discovery as candidate biomarker signatures. The source parallel multi-omics data are amplified in multiple data imputation processes (see Methods). Predictive imputation uses the dependencies between data dimensions to fill in missing data and replace data with uncertainty in different configurations. Stochastic imputation further increases the variety of generated data by helping existing data to form more variety of patterns. Using this framework, we can expose more data patterns to the accessible operational range of existing pattern recognition algorithms. Data imputation tools provide more frequent and more diversified opportunities for pattern discovery [19]. Thus, the hidden patterns are placed at shallower, easier to discover data entry locations for many imputation instances. This very-large-scale data amplification and integration process allows us to boost existing pattern discovery tools to solve more challenging information dependencies as candidate integrated biomarker signatures for further validation.

In addition to demonstrating the feasibility of parallel multi-omics, our prior studies also revealed new challenges in (a) integrating/synchronizing multiple datasets generated by using different platforms and annotation methods, and (b) extracting features from different data types to establish integrated biomarker signatures. Part of this challenge is the mapping of complex relationships across the multi-omics data because of the biological dependencies and interweaved interactions of the heterogeneous pathogenic processes that ultimately lead to T1D. Currently, computational tools for inter-omics synchronization (i.e., linking data across various omics sources) and intervention-based computational instruments are not yet good enough to adequately represent complex interaction systems such as human physiologic and pathologic processes. We propose that ultra-large-scale (and “ultra-deep”) exploration of the high-dimensional multi-omics dependencies would offer a transformative gateway towards a systematic understanding of the underlying pathological processes of T1D in at-risk subjects in comparison to healthy ones. However, multi-omics research in the T1D space is limited by the inherently small scale (in number of subjects and collection times) and breadth (in the coverage of hidden biomarkers) of relatively limited biological datasets obtained primarily from a vulnerable subject population (children). To address these limitations, we propose a novel approach of multiple imputation and amplification of existing biological datasets to virtually increase the number of subjects and expand the breadth in coverage of pattern discovery for enhanced identification of candidate biomarker signatures for further investigation (see Figure 1).

The theoretical framework of our proposed method expands the high-dimensional data processing capabilities of existing pattern discovery algorithms. The multi-omics data have an extremely unbalanced dimensional contrast between instance number (the number of subjects) and the omics landmark dimensions (the number of features/analytes). In high-dimension matrix analysis, the maximum rank of a matrix is equal to or smaller than the smallest dimension in the matrix (a brief introduction and mathematical derivation of this can be found in [12,13]). For example, for a two-dimensional matrix, the rank is smaller than either the row number or the column number. Statistically speaking, the rank of a stochastic matrix is correlated to the information content that can be extracted from it by analytical methods (theoretical analysis using symbolic derivations) or by algorithmic methods. Thus, only a small portion of the information content in the matrix can be utilized if the column number (landmark/feature number) is much larger than the row number (subject number), which often is the case in biological data. However, when the multi-omics data are amplified, and more rows are imputed/appended, the row and column numbers are better balanced towards a full utilization of the information content in the data. Notably, state-of-the-art “big data” bioinformatics tools perform better in discovering patterns when datasets have more instances/rows. However, increasing the instance/subject number means repeating the data collection, but this is a resource- and time-intensive process when scaled up and may not be feasible in biomedical studies where access to human subjects is limited and the time window for longitudinal analyses is not feasible, as is the case in children already diagnosed or at the risk of developing T1D. Hence, we propose the approach of data imputation and amplification to transform the “dimension-rich” biological parallel multi-omics data to “instance-rich” big data to empower existing bioinformatics tools and pattern discovery algorithms.

In this study, we explored whether combining a very-large-scale multiple imputation approach, such as those often employed in big data and artificial intelligence fields [14,15,16], with our integrated parallel multi-omics datasets obtained from a small number of subjects can meaningfully optimize the pattern discovery and facilitate the identification of T1D biomarker signatures by using existing data mining tools and software. Such imputations enlarge the biological datasets considerably and further enable the deployment of more powerful “big data” machine learning and pattern recognition tools that cannot be used in the original, much smaller datasets [17,18]. Thus, to demonstrate this novel concept, we employed a novel multiple imputation approach to our integrated quadra-omics dataset obtained from nine subjects with 2736 concentration datapoints (>2000 proteins, >300 miRNAs, >70 metabolites, and >40 lipids) determined in each subject, amplified it a thousand-fold (i.e., to the equivalent of 9000 virtual subjects), and assessed changes in the analytical capabilities and biomarker prediction power of the *Ingenuity Pathway Analysis (IPA)* software in the amplified dataset compared to the original. 

## 2. Materials and Methods

### 2.1. Sample Collection

Blood samples were collected at the Diabetes Research Institute of the University of Miami from consenting male/female subjects as previously described in detail [11]. In brief, ~20 mL of blood (in EDTA) was collected from subjects considered at high risk of T1D (*n* = 4) during routine visits as part of the ongoing TrialNet’s Natural History Study of the Development of Type 1 Diabetes (Pathway to Prevention Study) TN-01. During sample collection, one of the four high-risk subjects exhibited signs of the abnormal oral glucose tolerance test (OGTT) and was confirmed to have converted to a new-onset patient during a second OGTT and another sample collection two weeks later. Both samples were independently analyzed by multi-omics and constituted the new-onset group to avoid further reduction in the subject number. Blood samples from four healthy subjects were collected as part of another study approved by the IRB of the University of Miami (study number 11995-115). Plasma was obtained immediately after blood collection and stored at −80 °C until analysis. The reader is referred to Supplementary Table S1 in [11] for detailed demographic, serological, and other information of the subjects from whom samples were obtained. The studies under which samples were collected were conducted in accordance with the principles of the Declaration of Helsinki and consistent with the Good Clinical Practice guidelines of the International Conference on Harmonization. The protocol for the ancillary study, under which the quadra-omics analyses were performed, was approved by TrialNet and its IRB (study ID number 195).

### 2.2. Multi-Omics Analyses

Blood samples from all subjects were divided into four equal aliquots, which were independently subjected to proteomics, metabolomics, lipidomics, and transcriptomics (miRNAs) analyses performed as previously described in detail [11].

### 2.3. Algorithms for Multiple Imputation/Amplification of Multi-Omics

We employed proprietary algorithms for the multiple imputation and amplification of our parallel quadra-omics datasets (see the Data Availability Statement below explaining how to download). The system architecture on which this work was done is composed of four processing modules (algorithms) for (1) input data distribution and preprocessing analysis, (2) imputation pattern allocation, (3) data imputation/amplification, and (4) data storage. The input analysis module analyzes the data distribution in the source (original) multi-omics data to provide the data range (minimum and maximum values) and a measure of the data randomness (i.e., whether the data are concentrated on a few values or evenly distributed within the data range). The input analysis module ensures that the amplified data (in later steps) retain similar characteristics to the input source data. It also controls the level of randomness in the data imputation processes in the subsequent steps. This is important because the resulting data must have some differences among the amplified data entries (new “virtual” subjects) to avoid simple repetition of the existing data values. This gain in data diversity empowers the pattern discovery algorithms to identify patterns in the imputed/amplified datasets.

The input preprocessing module performs data cleaning and exception handling functions. The data cleaning algorithm identifies the locations of missing data entries and uses the median value of the same feature/analyte column to fill in the missing entries. The exception handling algorithm detects abnormalities in the source data and the errors in the preprocessing step and fills these exception locations with zero values to ensure that the source data meet the quality requirement of the amplification algorithm in the later steps. The data quality control functions of this module are essential for the subsequent data amplification algorithm to avoid amplifying and propagating errors in the source data. Notably, the zero values are different from the median values for each analyte, thereby allowing for their easy tracking without the need for additional location-finding masks in the imputed data. In addition, by definition, zero values are impossible for analyte expression levels and, thus, are easily distinguishable from the source data if/when exceptions or errors occur.

The next module, the imputation pattern allocation algorithm, expands the source data by first replicating them multiple times. The number of replications (imputation size) is mainly decided by the target depth of pattern discovery. A higher replication number means a higher amplification ratio, resulting in an imputed dataset with higher pattern discovery power. However, a larger imputed dataset demands more powerful and costly computation resources. Therefore, the depth of the target pattern discovery is determined based on empirical evaluation of prior similar data analyses with consideration of the available computational resources. For example, an amplification ratio of 10,000 means that each subject (instance of data) is repeated 10,000 times. The imputation pattern allocation algorithm first generates a random number matrix of the same size as the repeated source data. The random numbers are uniformly distributed between 0 and 1. Then a threshold is set to select a portion of the entries in the random number matrix. Entry locations with numbers smaller than the threshold are selected as the imputation locations. The selection threshold approximately equals the ratio of selection; for example, a selection threshold of 0.2 means that only entry locations with random numbers smaller than 0.2 will be selected. Because the random numbers are approximately evenly distributed between 0 and 1, around 20% of the random numbers are smaller than the 0.2 threshold. The entry locations are recorded as a selection mask matrix, and entry locations with random number values smaller than the selection threshold are assigned a “1” mask (“1” as an indicator for using imputed values). For all the instances (subjects) and all the feature (analyte) dimensions, the selection mask matrix forms a randomized pattern with selected locations scattered among all possible positions. In the next step (i.e., data imputation), the imputed data are inserted in the selected locations. This imputation pattern allocation algorithm forms the imputation location matrix in batches instead of testing each entry’s value individually. This improves the computational performance through the data structure vectorization. Processing multiple entries in batch (not using one by one iterations) allows matrix computation shortcuts and continuous memory allocation, which provides significant processing speed gains and better utilization of computation resources. The data imputation module (algorithm) fills the imputation locations as specified above in the imputation location matrix. The imputation values are computed as a random number within the data range of the corresponding feature dimensions. The imputation algorithm first calculates the minimum value, the maximum value, and the range from the data entries in each feature dimension of the source data matrix. For each imputation entry, a uniformly distributed number between 0 and 1 is calculated and multiplied by the range of the corresponding feature dimension. Then this value is added to the minimum value of the corresponding feature dimension as the imputed value. The algorithm first computes several descriptive statistics matrices of the same size of the amplified matrix. The algorithm uses a range matrix and a minimum value matrix to store the range values and the minimum value of each feature dimension, and then it computes a random number matrix filled with uniformly distributed random numbers. The imputation values are calculated using entry-wise multiplication and addition to form an imputation matrix with a value for each location of the amplified data matrix. For each entry, the imputation value is computed as the range multiplied by the random number, and then adding in the minimum value. The vectorized algorithm (batched version) can utilize many matrix-computation gains. Although this approach computes more entries than the number of the imputation entries, since an imputation value is still calculated for the entry locations retaining the source data, the processing speed gained from the vectorization far exceeds the reduction in processing speed, resulting from the iterative computation of redundant entries. Notably, the data amplification was performed uniformly on each subject group (i.e., with the same replication number) because their subject numbers were similar. For other datasets with extremely imbalanced instance/subject numbers in each category, the toolbox can specify different replication ratios for different categories to mitigate the imbalance, because pattern discovery algorithms usually work best when different classes/categories have approximately the same instance numbers.

The last and final step is the data storage algorithm, which organizes the imputed results into data blocks and has the capability to put each block into various file formats (e.g., .MAT, .CSV, and native HDF5). In our configuration, we put 10,000 instances (subjects) into one data block including all features/analytes for each subject. This configuration also compiles data entries with the same imputation ratio into the same data block. Moreover, multiple data blocks can also be stored into separate or single data files. The file format selection had two intuitive options in our implementation: (a) a MATLAB data format (.MAT) that utilizes the hierarchy in the data to increase the read and write speed, and (b) a comma-separated value format (.CSV) that allows easy exchange with other data segmentation options tailored to different distributed processing scenarios (computer clusters or cloud arrays). The MATLAB-format storage does not have limits on block size, while the CSV-format storage should be limited to 30 million entries (e.g., 10,000 entries/subjects, each with 3000 features/analytes). A larger data block is not recommended for the CSV-storage format to avoid system instabilities and server freezing.

### 2.4. Data Analysis

Data analysis was performed in the *Ingenuity Pathway Analysis (IPA)* software package (Qiagen Bioinformatics; Redwood City, CA, USA; https://www.qiagenbioinformatics.com/products/ingenuity-pathway-analysis, accessed on 27 August 2022; RRID:SCR_008653) [20]) using the *Canonical Pathway, Diseases and Functions, and Biomarker Prediction* modules. The same analyses were repeated in the original multi-omics datasets (which contained 2292 proteins, 328 miRNAs, 75 metabolites, and 41 lipids identified without exception in all nine analyzed samples) and the imputed/amplified (a thousand-fold) datasets which contained the same number of 2292 + 328 + 75 + 41 omics data per subject but for the equivalent of 9000 virtual subjects (4000 healthy controls, 3000 high risk of T1D, and 2000 new onset T1D), without any additional data curation based on fold-change. Three sets of amplified multi-omics datasets representative of lower (A1; 35%), intermediate (A2; 37.5%), and higher (A3; 40%) imputation levels were used around the ”golden ratio” of data imputation (38.2%:61.8%) [21]. Amplified datasets with increasing imputation level included a decreasing proportion of original values per feature in comparison to the source (original) dataset and relatively higher proportions of the algorithm-generated data, as described above. Amplified datasets A1, A2, and A3, respectively, contained 65%, 62.5%, and 60% of the original data. As in our previous study [11], *Canonical Pathway, Diseases and Functions*, and Biomarker Prediction analyses were iteratively performed for the high risk of T1D (HR) and new onset (NO) subject groups in the original and amplified multi-omics datasets independently or in combination (where indicated), and the findings in all four amplified datasets were compared to the corresponding original data (i.e., metabolomics, proteomics, lipidomics, and transcriptomics). Data plots and comparative analyses were generated/performed in GraphPad Prism version 9.3.1 for Windows (GraphPad Software, San Diego, CA, USA, www.graphpad.com, accessed on 27 August 2022). 

## 3. Results

We previously demonstrated the potential of parallel multi-omics in identifying candidate integrated biomarker signatures owing to the high dimensionality of the data, but we also identified challenges in the synchronization and integration of the multi-omics data obtained through different analytical methods and from a limited number of subjects [11]. In the present study, we investigated whether a novel computational approach of data imputation and amplification to virtually increase the number of subjects will mitigate this limitation and enhance the analytical capabilities of *the* *Ingenuity Pathway Analysis (IPA)* software in identifying novel features and patterns as potential integrated biomarker signatures for further investigation (Figure 1). Starting from our original parallel quadra-omics datasets collected from human subjects at high risk of T1D (HR, *n* = 3), recently diagnosed (new-onset, NO, *n* = 2) and healthy controls (*n* = 4) that contained 2292 protein, 328 miRNA, 75 metabolite, and 41 lipid datapoints measured without exception in all subjects [11], we generated multiple corresponding thousand-fold amplified datasets using the proprietary imputation/amplification method described above. The integrated amplified datasets contained the same number of omics data per subject (i.e., 2292 + 328 + 75 + 41) but for the equivalent of 9000 virtual subjects. Three amplified datasets representing lower, intermediate, and higher imputation levels within the range of the “golden ratio” of data imputation were then analyzed using the Canonical Pathway, Diseases and Functions, and Biomarker Prediction modules of *IPA*, and results were compared to those obtained in the non-amplified original data. 

### 3.1. Comparative Enrichment Analyses for Canonical Pathways

**Figure 2:**
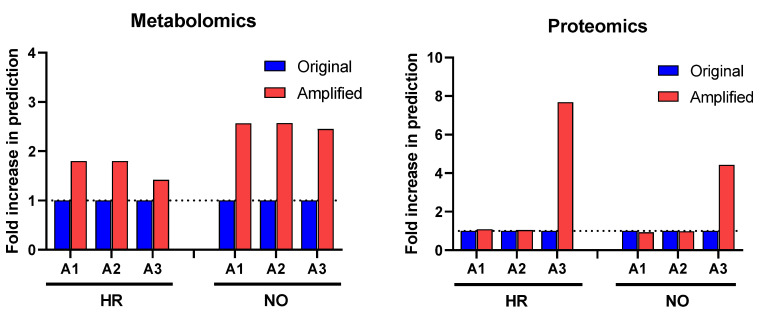
Change in prediction yields of canonical pathways identified in the amplified (A1, A2, and A3) vs. original proteomics and metabolomics datasets by *IPA* based on published literature available in its knowledge-database as of the time of performing the analysis (https://go.qiagen.com/IPA-transcriptomics-whitepaper, accessed on 27 August 2022). Numbers of canonical pathways predicted in the amplified datasets (red) are shown as fold-change (increase above dotted line) in comparison to the original datasets (blue) for the high-risk (HR) and new-onset (NO) subject groups. Comprehensive lists of these canonical pathways are presented in Supplementary Table S1A,B (for metabolomics) and Supplementary Table S2A,B (for proteomics) in HR and NO subject groups.

Our first approach in interrogating the amplified datasets (A1–A3) compared to the original data focused on canonical pathways, which are established cell signaling and metabolic pathways with well-characterized intermediaries. Enrichment analyses for canonical pathways were performed using *IPA* in all three amplified quadra-omics datasets independently and were compared to those done in the corresponding original non-amplified data. Similar to our prior studies [11], current analyses in the transcriptomics and lipidomics datasets had extremely limited yields that rendered no statistically significant predictions of canonical pathways in the amplified or original datasets of either subject group. Analyses in the amplified proteomics and metabolomics datasets of the high risk and new onset subject groups, respectively, identified increased numbers of canonical pathways compared to the corresponding original data (Figure 2). Specifically, analysis in metabolomics amplified datasets A1, A2, and A3 with increasing imputation levels, respectively, showed an approximate 2-fold increase in canonical pathways predicted/identified in the high risk (HR) and new onset (NO) subject groups. Similar analyses in the proteomics datasets found no difference in amplified datasets A1 and A2 versus the original data, but a marked 4- and 8-fold increase was identified in amplified dataset A3 for the new-onset and high-risk groups, respectively. Supplementary Tables S1A,B and S2A,B show the comprehensive lists of canonical pathways identified/predicted by these analyses. Table 1 shows a condensed list of pathways with involvement in inflammatory processes and direct links to T1D based on published literature. 

### 3.2. Comparative Enrichment Analyses for Diseases and Functions

#### 3.2.1. Enhanced Prediction of T1D-Relevant Immune Functions in Multi-Omics Datasets Independently

**Figure 3:**
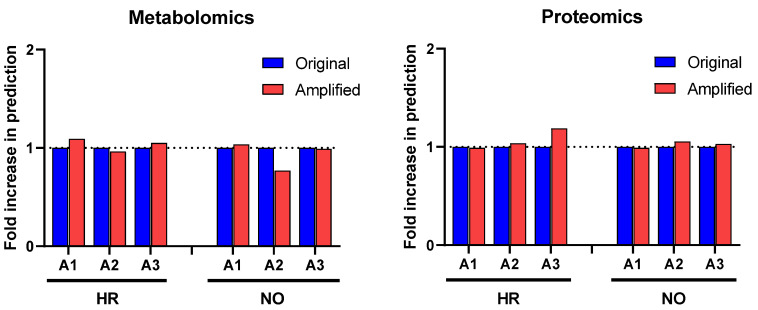
Comparison of prediction yields of diseases and functions identified in the amplified and original proteomics and metabolomics datasets by *IPA* based on published literature available in its knowledge database as of the time of performing the analysis (https://go.qiagen.com/IPA-transcriptomics-whitepaper, accessed on 27 August 2022). Total numbers of diseases and functions predicted in the amplified datasets are shown as fold-change (increase or decrease) in comparison to the original data for the HR and NO subject groups.

**Figure 4:**
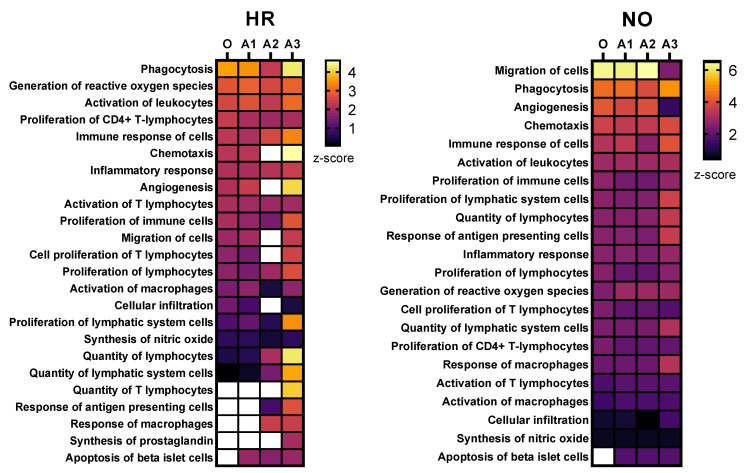
Heat maps showing selected immune functions identified in the integrated amplified proteomics and metabolomics datasets (A1, A2, and A3) in comparison to the original data (O) for both the HR and NO subject groups. Heat maps were generated based on the average *z*-score assigned by *IPA* to each predicted function in the metabolomics and proteomics datasets for both amplified and original datasets. Functions were sorted by the *z*-score for the original dataset. The *z*-score is a statistical measure that accounts for the directional effect of change and the magnitude of its impact on the affected disease/function (https://go.qiagen.com/IPA-transcriptomics-whitepaper, accessed on 27 August 2022). Functions were selected based on their direct involvement in inflammatory and immune responses and, hence, as relevant to T1D (see Supplementary Tables S3 and S4 for complete lists of identified functions).

Further enrichment analyses performed in *IPA* for diseases and functions were performed independently in all three amplified (A1, A2, and A3) and original metabolomics, proteomics, transcriptomics, and lipidomics datasets of the HR and NO subject groups. Analysis in the transcriptomics datasets yielded some predictions but without relevance to T1D (see Figure S1). Analysis in the lipidomics datasets did not yield any predictions (not shown). Analyses in amplified metabolomics and proteomics datasets A1, A2, and A3, respectively, showed a slight increase in the numbers of diseases and functions identified versus the corresponding original data (Figure 3). Comprehensive lists of diseases and functions identified in all amplified and original datasets and consolidated (integrated) from the metabolomics and proteomics independent analyses are shown in Supplementary Table S3A,B for the T1D HR and NO subject groups, respectively. Despite the modest impacts of data amplification on the global prediction yields for diseases and functions, focusing the analysis on immune/inflammatory processes enhanced the prediction power in the integrated proteomics and metabolomics datasets of immune functions directly implicated in T1D and its pathogenesis, as shown in Figure 4. This highlights the biological relevance of the enhanced predictions rather than merely amplifying them in a general way. While all the predicted T1D-related immune functions have known involvement in inflammation, several had higher *z*-score values in the amplified datasets when compared to the original. Notably, “*apoptosis of beta islet cells*”, which is directly implicated in T1D pathogenesis, was exclusively predicted in amplified datasets but not in the original data of both subject groups. There were also other immune functions exclusively predicted in amplified dataset A3 of the HR subjects, which are known to increase susceptibility to inflammation and the associated islet damage in T1D (e.g., “*quantity of T lymphocytes”, “response of macrophages”, and “synthesis of prostaglandin*”) [53,54,55,56]. 

#### 3.2.2. Enhanced Prediction of Immune and Inflammatory Diseases and Functions in Amplified Integrated Multi-Omics Datasets

**Figure 5:**
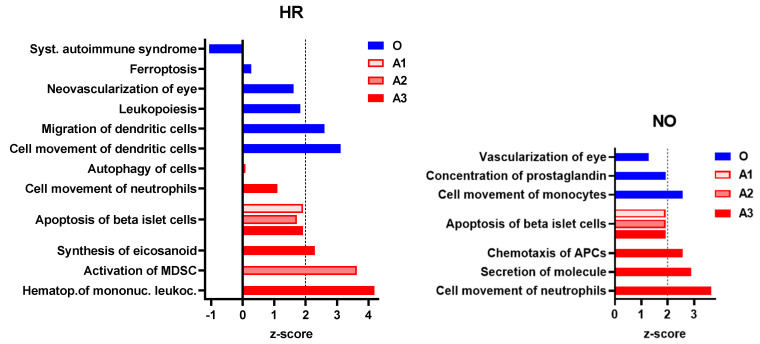
Immune and inflammatory diseases and functions exclusively predicted in the amplified (A1, A2, and A3) or original (O) integrated proteomics–metabolomics datasets for both the HR and NO subject groups (also see Figure 4 and Supplementary Table S3A,B for complete lists of predicted diseases and functions). Bar graphs show the *z*-scores, where positive values indicate predicted activation and negative values the inhibition of the specific immune disease and/or function (*z*-score values ≤ −2 or ≥2 are considered significant; https://go.qiagen.com/IPA-transcriptomics-whitepaper, accessed on 27 August 2022).

To further evaluate whether our data imputation approach enhanced the ability of *IPA* in identifying T1D-related pathogenic processes that were not identified in the original data, we performed enrichment analysis for diseases and functions in all three amplified integrated proteomics–metabolomics datasets (A1, A2, and A3) in comparison to the corresponding original integrated data. These analyses identified exclusively in the amplified datasets new immune processes/functions that were significantly impacted, albeit to various degrees, in the T1D HR and NO subject groups in comparison to healthy subjects (Figure 5). The analyses also exclusively identified other immune functions in the original data but, contrary to expectations, “*systemic autoimmune syndrome*” was assigned negative z-score values, suggesting its reduced propensity in the high-risk subject group. Notably, “*systemic autoimmune syndrome*” was not predicted in any of the amplified data, whereas, on the other hand, “*apoptosis of islet beta cells*” was exclusively predicted in all three amplified datasets of both HR and NO subject groups, which is consistent with the expectation that subjects at very high risk of developing T1D are likely to have ongoing beta cell destruction, just as those recently diagnosed.

### 3.3. Biomarker Prediction in the Amplified Versus Original Proteomics and Metabolomics Datasets

**Figure 6:**
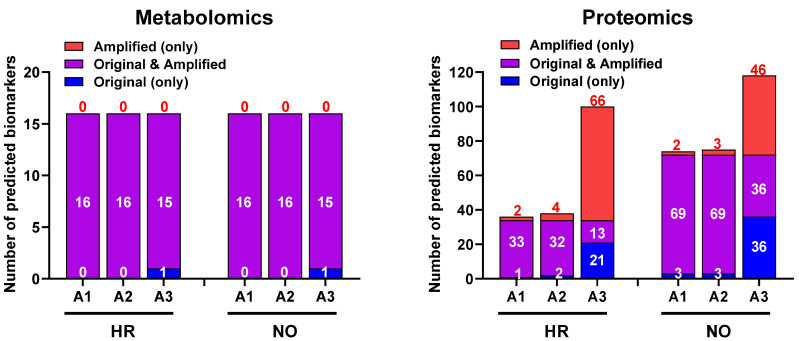
Bar graphs showing the numbers of candidate biomarkers predicted in the amplified datasets only (A1, A2, A3; red), the original data only (blue), or in common in both (purple) for the HR and NO subject groups. Numbers of predicted biomarkers belonging to each group are marked within or above the corresponding bars.

**Figure 7:**
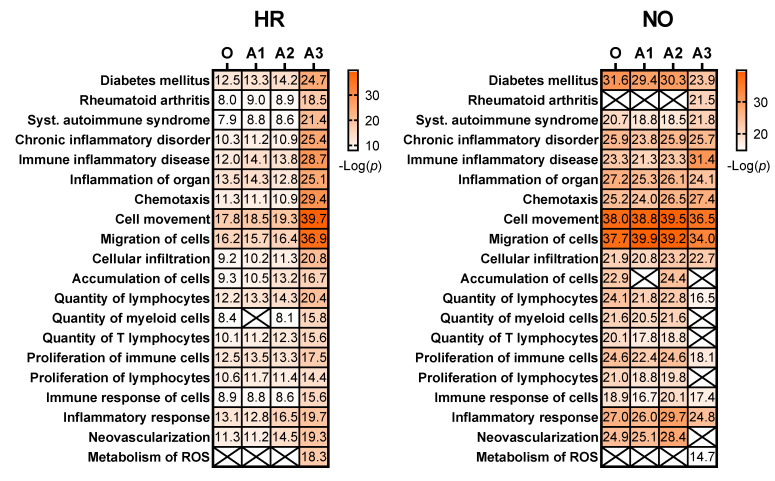
Heat maps showing selected diseases and functions that are enriched for immuno-inflammatory processes associated with candidate biomarkers predicted in the amplified integrated proteomics–metabolomics datasets (A1, A2, A3) in comparison to the original data (O) for the HR and NO subject groups. Values shown within the heat map cells correspond to the negative Log of the *p*-value assigned by *IPA* to each prediction based on Fisher’s tests. Cells marked with an X represent no prediction by *IPA* as of the date of analysis. See Supplementary Table S4A,B for complete lists of biomarker candidates associated with each predicted disease and function.

We next evaluated whether our data imputation approach improves the biomarker prediction power of *IPA* in amplified datasets compared to the original data. We performed the analyses in the proteomics and metabolomics datasets independently because such predictions in the integrated quadra-omics datasets are currently not possible in *IPA*. The analyses were performed in the proteomics and metabolomics datasets for each subject group (high-risk and new-onset T1D) both separately (Figure 6) and combined (Figure 7). For metabolomics, the results showed relatively similar predictions of candidate biomarkers in the amplified and original datasets. There were 16 predicted biomarker candidates in total, which were common to the amplified and original datasets, except for A3, where only 15 biomarkers were predicted (Figure 6; left panel). Similar analyses in the proteomics amplified datasets A1 and A2 also yielded candidate biomarkers of comparable numbers to the original data; however, there was a significant increase in the number of candidate biomarkers predicted exclusively in amplified dataset A3 for both the HR and NO subject groups (66 and 46, respectively) (Figure 6; right panel). Additional examination of diseases and functions associated with the candidate biomarkers predicted in the integrated datasets for each subject group showed enrichment for immuno-inflammatory processes with often increased significance in the amplified datasets, especially in A3 (Figure 7). Complete lists of biomarker candidates predicted in association with each disease and function in the amplified and corresponding original datasets for the high-risk and new-onset subject groups are shown in Supplementary Table S4A,B, respectively.

## 4. Discussion

We are developing a framework to allow the use of “big data” tools and pattern discovery instruments for the identification of biomarker signatures of T1D from parallel multi-omics human datasets by deploying very-large-scale multiple imputation of the original data, where the number of samples available for analysis is typically limited. A schematic depiction of the workflow to identify such biomarkers is provided above in Figure 1. This work builds on our recent studies that demonstrated the feasibility of parallel multi-omics and its potential for the identification of integrated T1D biomarkers that are currently needed desperately [11]. Our recent studies also identified challenges in the post-acquisition synchronization and integration of multi-omics data, which were obtained by various analytical methods and annotated differently, as is currently the only way possible. 

As noted earlier, current computational tools for inter-omics synchronization and intervention-based mechanisms are currently underdeveloped for complex interaction systems such as human pathologic processes that lead to diseases such as T1D. However, we posit that collecting and analyzing multi-omics data from multiple streams is akin to the approaches of the parallel linguistic corpus in natural language machine translation research, and where significant progress has been made [57], and data integration tools available in that domain can be adapted to aid the discovery of integrated T1D biomarker signatures; but the complex mapping of relationships in the high-dimensional multi-omics data is dependent on the interconnected biological interactions and signaling events underlying the T1D pathogenesis. Therefore, a parallel approach, where multiple tracks of data related to the same source (i.e., single patient or multiple patients at the same disease stage) in such “multi-language” datasets facilitate the extraction of information by exposing dependencies in multi-track formats. Similar parallel natural language processing in text datasets of multiple languages and algorithmic/computational development have triggered the artificial intelligence (AI) revolution in language machine translation (e.g., *Aligned Hansards of the 36th Parliament of Canada* and *European Parliament Proceedings Parallel Corpus* [58]). 

Currently, most integration approaches use a one-go deterministic pattern discovery process. In the present study, we implemented a novel multiple imputation approach to repeat such a process thousands or millions of times, with stochastic mechanisms appended to individual processes. The imputation process allows the data to be fully utilized, where data instances using different imputation mask patterns (as described in Methods) allow the pattern discovery algorithms to explore the data from different angles. This capability is especially beneficial for exposing hidden information in the high-dimensional biological data and increasing the pattern discovery power of current algorithms and amplifying their analytical capabilities with the potential to solve demanding and fundamental challenges in multi-omics data integration/synchronization. Notably, one experiment usually cannot capture the complexity of interactions across multiple data sources (e.g., features, synchronized/aligned data blocks). Alternatively, many parallel processes (e.g., 10 million to 10 billion correlated high-dimensional pattern discovery processes) will provide more opportunities for more data segments or dimensions to connect to each other, where each process will help expose part of the data to the machine learning and data science algorithms. This approach is somewhat like the multiple imputation frameworks from missing data technology, but with predictive analyses at a larger scale compared to conventional approaches. Importantly, without this iterative process of pattern exposure (or data amplification), similar patterns are usually buried deeply under other dependencies and are difficult to identify. Thus, our proposed framework of multiple imputation can contribute toward solving the complex and challenging problem of mapping the relationships in the high-dimensional integrated/synchronized parallel multi-omics datasets for better identification of unique patterns as candidate biomarker signatures for further validation experimentally and clinically (Figure 1). 

Another problem in biological data is their inherent intra- and inter-subject variability, as commonly is the case in clinical data from patients, which is further exacerbated by the issues of accurate quantitation of very low concentration analytes and small sample sizes (i.e., number of subjects). When there are missing data values for certain individuals or from a data source in an integrated dataset, imputation methods are often used to iteratively fill the blanks in the data. This missing data mitigation framework empowers the machine learning algorithms towards more diverse inter-data connections. Consistently, we also expect that randomly hiding (or masking-off) parts of the data, and using multiple imputations to fill these “gaps”, will further enable the discovery of more patterns than in a one-go process. Essentially, this approach extends the missing data technology into a framework of data/pattern amplification by hiding data using different mask patterns and running many related pattern discovery processes. We expect such large-scale data integration and imputation/amplification to further push the data processing and pattern discovery pipelines towards large-scale complex-system levels for decoding the complex mapping relationships inside the parallel multi-omics datasets and to ultimately uncover novel patterns that may prove useful as T1D biomarker signatures in clinical applications. Importantly, this will enable more efficient utilization of multi-omics datasets obtained in precious biological samples from limited subject populations, as is the case with children considered at various levels of risk of developing T1D and from whom obtaining samples is more challenging.

To evaluate the above theoretical framework, we applied the multiple imputation approach to our existing quadra-omics dataset that was obtained from nine subjects and amplified a thousand-fold to the equivalent of 9000 subjects and assessed how this changed the analytical capabilities and biomarker prediction power of the widely used *Ingenuity Pathway Analysis (IPA)* software. As was done before [11], the Diseases and Functions analyses yielded a wealth of information that was not much different between the original and the amplified data in terms of quantity (i.e., total numbers) of predictions (Figure 3). However, restricting the scope of predictions to immuno-inflammatory processes identified immune functions with increasing significance in the amplified datasets compared to the corresponding original data from both the T1D high-risk and new-onset subject groups (Figure 4). This increased prediction power was most pronounced in amplified dataset A3, which had the highest level of imputation (40%) among those we tested here around the ”golden ratio” of data imputation (i.e., 38.2%:61.8%) [21]. Several immune functions with direct relevance to T1D (e.g., “*activation of antigen presenting cells, macrophages, and T cells”; “biosynthesis of prostaglandins”; and “beta cell apoptosis*”) were exclusively identified in amplified datasets A2 and A3 of the T1D high risk subjects, but not in the original data. 

Of special interest for validating the biological relevance of our data imputation/amplification framework were the analyses of *canonical pathways*, since they represent well-characterized biochemical/signaling pathways and cellular events that are established in health and various diseases based on well-documented scientific literature. Table 1 showed several canonical pathways with direct involvement in autoimmune T1D that were exclusively predicted with statistical significance in the metabolomics and proteomics A3 datasets of both subject groups. Among them were the arginine-dependent production of nitric oxide and reactive oxygen species (ROS) in macrophages [38,39] and the increased signaling of proinflammatory cytokines/chemokines and Rho kinases [26,27,30,31,40,41]. Moreover, sphingosine-1-phosphate (S1P), oncostatin M (OSM), paxillin (PXN), and human leukocyte antigen-F adjacent transcript 10 (FAT10; aka ubiquitin D, UBD) were also exclusively identified in the amplified data of the T1D high-risk subject group, which was in agreement with published literature proposing these molecules (i.e., S1P, OSM, PXN, and FAT10) as biomarkers for increased risk of T1D [36,37,42,46]. Notably, none of the above pathways nor S1P, OSM, PXN, and FAT10 were identified/predicted to be significantly altered in the original data, which highlighted the improvement in the analytical power of *IPA* through our data imputation approach. 

Moreover, the *IPA*’s Biomarker Prediction module provided a host of biomarker candidates from the amplified datasets that were relevant to various immune and autoimmune conditions including T1D. Table 2 below shows the consolidated lists of biomarker candidates predicted in the original and the amplified metabolomics/proteomics datasets from the T1D high-risk and new-onset subject groups (also see Supplementary Table S4A,B for comprehensive lists of predicted biomarker candidates in association with other immune diseases and functions). As is shown in Figure 6 and Figure 7, the biomarker prediction power of *IPA* was most enhanced in amplified dataset A3 in comparison to the original, and most biomarker predictions in this specific analysis module were based on the proteomics data. We do not know the specific reason for the limited biomarker predictions in the metabolomics datasets, whether original or amplified, but this might in part be due to the compression in the metabolites abundance values in our prior analyses in association with the tandem mass tagging method used to barcode the samples [11,59]. Nonetheless, these predictions provided promising candidate biomarker signatures that can be further validated in future studies, as we discussed above and depicted in Figure 1. 

**Figure 8:**
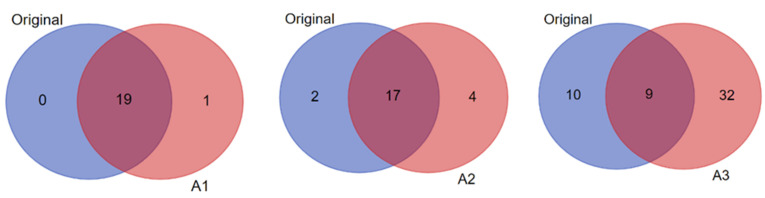
Pairwise comparisons (shown as Venn diagrams) between the original and the amplified datasets A1, A2, and A3 of the T1D high-risk subject group showing the numbers of biomarker predictions made by *IPA* in the context of “diabetes mellitus” in common between each pair (i.e., original versus A1; original versus A2; and original versus A3), and the numbers of unique predictions made in each dataset (i.e., original versus amplified).

Notably, while some of the candidates within each biomarker signature were predicted in common in the original and amplified datasets (see Supplementary Table S4A,B), the number of unique predictions in the amplified data compared to the original increased progressively with the increased level of data imputation/amplification (Figure 6). For example, when considering candidate biomarkers predicted in the context of “diabetes mellitus” in the T1D high-risk subject group, A3 yielded the highest total number and unique predictions in comparison to the original data and more than A1 and A2 as well (Figure 8). The statistical significance of the predictions was also enhanced with the increased data imputation level, especially in the T1D high-risk subject group (Table 2; also see Figure 7). Importantly, while the quantity of biomarker predictions increased in the amplified datasets, the quality of the predictions was also enhanced in terms of disease relevance. A close examination of the 19 candidate biomarkers predicted by *IPA* in the context of “diabetes mellitus” in the original dataset identified 15 biomarkers with direct connections to autoimmune T1D, as opposed to type 2 diabetes (T2D). This number, however, increased to 28 such biomarker candidate predictions in A3, and 23 of these predicted biomarkers were unique to A3 and not predicted in the original dataset. This demonstrated the enhanced prediction power of *IPA*’s algorithms in association with our novel approach of parallel multi-omics data imputation/amplification. 

## 5. Summary and Conclusions

In summary, the above analyses showed improved quantitative and qualitative abilities of *IPA* software to identify T1D relevant pathways and associated biomarker candidates in the imputed/amplified datasets compared to the original data obtained from a small number of subjects. An intuitive interpretation of this increased pattern discovery capability is that imputed/amplified data enable more biologically and disease-relevant patterns to emerge by bridging key data points in originally separated/fragmented patterns. Our current findings also indicated that small perturbations in the data helped the pattern discovery algorithm(s) to unveil hidden data dependencies, much like how simple additional tracing highlights the contours of a concept in an abstract painting. Moreover, the data imputation procedures we employed here enabled us to explore multiple data amplification ratios to generate multiple versions of amplified datasets for further exploration from different perspectives (computationally speaking) and, thereby facilitated the “full” utilization of the rich information embedded in the original high-dimensionality biological data. This preliminary work showed that data imputation at a level close to the “golden ratio” (38.2%:61.8%) was optimal within our quadra-omics datasets, which is consistent with multiple imputation frameworks from missing data technology in other applications [97,98,99]. Next steps in this project will expand the distributed storage and processing capabilities of our data amplification/imputation algorithms to achieve “big data” level data imputation and further enable larger-scale decoding of the complex relationships inside expanded biological parallel multi-omics datasets with additional subjects using more powerful “big data” machine learning and pattern discovery tools.

Based on the current preliminary work, we conclude that our novel approach of data imputation and amplification of limited, yet high-dimensionality parallel multi-omics datasets can be used to increase the analytical capabilities and the predictive range of existing algorithms and data mining instruments, and to potentially enable the deployment of more powerful “big data” machine learning and pattern recognition tools to enhance the identification of promising disease-specific biomarkers and biomarker signatures that may ultimately aid in the diagnosis and treatment of autoimmune T1D among other human conditions. 

## Figures and Tables

**Table 1:** Selected canonical pathways identified/predicted in the original and amplified proteomics and metabolomics datasets for the HR and NO subject groups. Selection was based on involvement in inflammatory processes, such as cytokine and chemokine signaling and immune cell functions, and subsequent validation in published literature with direct relevance to T1D. Shown are the values of −log(*p*) for each identified/predicted canonical pathway in each dataset, and only those with at least one significant prediction (i.e., −log(*p*) > 1.3 or *p* < 0.05) are shown. When significance was reached in one of the datasets (original or amplified), parallel non-significant predictions with −log(*p*) values < 1.3 are shown in gray color to highlight differences. Blank means no prediction.

			−log(*p*-Value) *				
		Canonical Pathways	Original	A1	A2	A3	References
**T1D High-Risk (HR)**	**Proteomics**	CCR3 Signaling in Eosinophils	0.206	0.201	0.201	4.03	[22,23]
		Complement System	0.631	0.623	0.625	5.69	[24,25]
		CXCR4 Signaling				5.53	[26,27]
		Fcγ Receptor-Mediated Phagocytosis in Macrophages	0.833	0.821	0.824	9.16	[28]
		FcγRIIB Signaling in B Lymphocytes		0.333	0.333	2.39	[29]
		IL-12 Signaling and Production in Macrophages	0.592	0.582	0.583	4.65	[30,31]
		IL-15 Production	2.71	2.67	3.54	7.42	[32,33]
		IL-7 Signaling Pathway		0.361	0.361	1.97	[34,35]
		Oncostatin M Signaling		0.567		1.73	[36]
		Paxillin Signaling	0.266	0.261	0.262	7.39	[37]
		Production of Nitric Oxide and ROS in Macrophages	0.398	0.389	0.39	2.67	[38,39]
		RHOA Signaling	1.22	1.2	1.2	7.37	[40,41]
		Sphingosine-1-Phosphate Signaling	0.237	0.232	0.233	3.23	[42]
	**Metabolomics**	Arginine Biosynthesis IV	3.79	12.2	5.4	5.39	[38,43]
		Citrulline–Nitric Oxide Cycle	1.35	6.83	3.11	3.1	[43,44]
		Stearate Biosynthesis I (Animals)	1.16	4.02	2.67	2.67	[45]
		FAT10 Signaling Pathway	1.04	1.84	2.29	2.29	[46]
**T1D New-Onset (NO)**	**Proteomics**	14-3-3-Mediated Signaling				3	[47]
		CCR3 Signaling in Eosinophils				4.63	[48]
		CXCR4 Signaling	0.259			4.82	[26,27]
		Fcγ Receptor-Mediated Phagocytosis in Macrophages	0.666	0.664	0.325	3.36	[28]
		FcγRIIB Signaling in B Lymphocytes			0.375	3	[29]
		Oncostatin M Signaling	0.282	0.281	0.284	2.45	[36]
		PAK Signaling				6.01	[49,50]
		Phospholipases	0.975	1.56	0.983	3.7	[51,52]
		RHOA Signaling	0.793	0.45	0.457	8.12	[40,41]
		Sphingosine-1-Phosphate Signaling	0.475	0.474		4.48	[42]
	**Metabolomics**	Arginine Biosynthesis IV	2.92	12.2	5.4	5.39	[38,43]
		Citrulline–Nitric Oxide Cycle	1.45	6.83	3.11	3.1	[43,44]
		Stearate Biosynthesis I (Animals)	1.26	4.02	2.67	2.67	[45]
		FAT10 Signaling Pathway	1.09	1.84	2.29	2.29	[46]

* −log(*p*) values > 1.3 are significant (i.e., *p* < 0.05). Non-significant predictions (with −log(*p*) values < 1.3) are shown in gray to highlight differences among the various datasets. Blank means no prediction.

**Table 2:** Consolidated lists of candidate biomarkers predicted by *IPA* in the context of “diabetes mellitus” (top row) in the original and amplified datasets of the T1D high-risk subject group without any further data filtration or curation based on expression fold-change, and those among them that were confirmed as relevant to T1D based on published references (bottom row).

High-Risk T1D (HR)		Original		A1		A2		A3
	*p*	Biomarker Name *	*p*	Biomarker Name *	*p*	Biomarker Name *	*p*	Biomarker Name *
**Diabetes Mellitus**	3.53 × 10^−13^	APOA2, APOE, CD44, CETP, GPNMB, IGF1, IGFBP2, JAG1, L1CAM, LDLR, MEP1B, MMP14, MMP2, MMP9, PTGDS, PTPRC, SELL, SFTPD, VCAM1	4.69 × 10^−14^	APOA2, APOE, CD44, CETP, GPNMB, IGF1, IGFBP2, IGHM, JAG1, L1CAM, LDLR, MEP1B, MMP14, MMP2, MMP9, PTGDS, PTPRC, SELL, SFTPD, VCAM1	6.1 × 10^−15^	ANXA1, APOA2, APOE, CD44, CETP, FGFR1, GPNMB, IGF1, IGFBP2, IGHM, JAG1, L1CAM, LDLR, MEP1B, MMP14, MMP2, MMP9, PTGDS, SELL, SFTPD, VCAM1	1.91 × 10^−25^	ACE, ADK, AKT1, APOA1, APOA4, APOB, APOC1, CASP3, CCL5, CD36, CETP, CR2, CXCL12, EGFR, FADD, FAS, FGFR1, GFAP, GSTO1, GSTP1, HPSE, HSPB1, IGF1, IGF2, IGHM, IL18, L1CAM, MASP2, MMP14, MMP9, MSTN, PCSK9, PDE5A, PON1, PTGDS, PTPRC, RETN, SFTPD, SOD1, SRC, VCAM1
T1D **	n/a	APOA2 [60], APOE [61], CD44 [62], CETP [63], GPNMB [64], IGF1 [65], IGFBP2 [66], JAG1 [67], LDLR [68], MEP1B [69], MMP2 [70], MMP9 [70], PTPRC [71], SELL [72], VCAM1 [73]	n/a	APOA2 [60], APOE [61], CD44 [62], CETP [63], GPNMB [64], IGF1 [65], IGFBP2 [66], IGHM [74], JAG1 [67], LDLR [68], MEP1B [69], MMP2 [70], MMP9 [70], PTPRC [71], SELL [72], VCAM1 [73]	n/a	ANXA1 [75], APOA2 [60], APOE [61], CD44 [62], CETP [63], FGFR1 [76], GPNMB [64], IGF1 [65], IGFBP2 [66], IGHM [74], JAG1 [67], LDLR [68], MEP1B [69], MMP2 [70], MMP9 [70], SELL [72], VCAM1 [73]	n/a	ACE [77], APOA1 [60], APOB [78], CASP3 [79], CCL5 [80], CETP [63], CR2 [81], CXCL12 [82], FADD [83,84], FAS [83,85], FGFR1 [76], GFAP [86], HPSE [87], HSPB1 [88], IGF1 [65], IGF2 [65], IGHM [74], IL18 [89], MASP2 [90], MMP9 [70], MSTN [91], PCSK9 [92], PDE5A [93], PON1 [94], PTPRC [71], RETN [95], SRC [96], VCAM1 [73]

* Biomarker names correspond to the associated gene names. Shown *p*-values correspond to averaged values provided by *IPA* for the consolidated biomarker candidates predicted in the context of “diabetes mellitus” (inclusive of T1D and T2D) in the proteomics and metabolomics datasets independently, since *IPA* cannot currently perform biomarker predictions in integrated multi-omics data. ** Biomarkers confirmed as relevant to T1D based on the published references cited next to each candidate biomarker. Candidate biomarkers highlighted in red were exclusively predicted in the amplified datasets.

## Data Availability

The original multi-omics datasets previously generated and used in the new analyses in the current study have been deposited in the following publicly accessible repositories: the NIH Common Fund’s National Metabolomics Data Repository (NMDR; www.metabolomicsworkbench.org) [100], accession #s ST001690 (doi:10.21228/M8B123) for the metabolomics dataset and ST001642 (doi:10.21228/M8ZX18) for the lipidomics dataset; the PRIDE database of ProteomeXchange (https://www.ebi.ac.uk/pride/, accessed on 27 August 2022)), accession # PXD023541 for the proteomics dataset; and the Harvard Dataverse repository (doi.org/10.7910/DVN/A2OU24) for the transcriptomics dataset. The amplified datasets (A1–A3) containing the combined quadra-omics data and the code for the proprietary algorithms used to generate them can be freely accessed and downloaded in R, Python, and MATLAB scripts through the following link: https://miamiedu-my.sharepoint.com/personal/mabdulreda_miami_edu/_layouts/15/onedrive.aspx?id=%2Fpersonal%2Fmabdulreda%5Fmiami%5Fedu%2FDocuments%2FAbdulreda%20Lab%2FAmplified%20quadra%2Domics%20datasets%2BCode&ga=1, accessed on 27 August 2022.

## Supplementary Materials

The following supporting information can be downloaded at: https://www.mdpi.com/article/10.3390/biom12101444/s1, Figure S1: Comparison of prediction yields of immuno-inflammatory diseases and functions in the amplified and original small transcriptomics (miRNAs) datasets for the T1D high-risk (HR) and new-onset (NO) subject groups; Table S1A,B: Complete lists of canonical pathways in the original and amplified metabolomics datasets (A1, A2, and A3) of the (S1A) HR and (S1B) NO subject groups; Table S2A,B: Complete lists of canonical pathways in the original and amplified proteomics datasets (A1, A2, and A3) of the (S2A) HR and (S2B) NO subject groups; Table S3A,B: Complete lists of immuno-inflammatory diseases and functions enriched in the integrated multi-omics datasets for (S3A) HR and (S3B) NO subject groups; Table S4A,B: Lists of consolidated biomarkers identified by *IPA*’s Biomarker Prediction module in the context of the selected diseases and functions in the proteomics and metabolomics amplified and original datasets for the (S4A) HR and (S4B) NO subject groups.
