# Supplementary material for: Exploring Computational Data Amplification and Imputation for the Discovery of Type 1 Diabetes (T1D) Biomarkers from Limited Human Datasets
Source: Biomolecules. 2022 Oct 9;12(10):1444. doi: 10.3390/biom12101444 (PMC9599756; doi:10.3390/biom12101444)
Supplement: Supplementary file 1 [file biomolecules-12-01444-s001.zip › biomolecules-1919603-supplementary.pdf]

biomolecules

Supplementary Materials

Exploring Computational Data Amplification and Imputation for the Discovery of Type 1 Diabetes (T1D) Biomarkers from Limited Human Datasets

Oscar Alcazar [1,†], Mitsunori Ogihara [2,†,*], Gang Ren [2,†,*], Peter Buchwald [3,*] and Midhat H. Abdulreda [4,*]

[1] Diabetes Research Institute, University of Miami Miller School of Medicine, Miami, FL, 33136, USA
[2] Institute for Data Science and Computing, Department of Computer Science, University of Miami, Coral Gables, FL, 33146, USA
[3] Diabetes Research Institute, Department of Molecular and Cellular Pharmacology, University of Miami Miller School of Medicine, Miami, FL, 33136, USA
[4] Diabetes Research Institute, Department of Surgery, Department of Microbiology and Immunology, Department of Ophthalmology, University of Miami Miller School of Medicine, Miami, FL, 33136, USA
* Correspondence: m.ogihara@miami.edu (M.O.); gxr467@miami.edu (G.R.); pbuchwald@miami.edu (P.B.); mabdulreda@miami.edu (M.H.A.); Tel.: +1-30-5284-2308 (M.O.); +1-30-5243-1649 (G.R.); +1-30-5243-9657 (P.B.); +1-30-5243-9871 (M.H.A.)
† These authors contributed equally to this work.

Citation: Alcazar, O.; Ogihara, M.; Ren, G.; Buchwald, P.; Abdulreda, M.H. Exploring Computational Data Amplification and Imputation for the Discovery of Type 1 Diabetes (T1D) Biomarkers from Limited Human Datasets. *Biomolecules* **2022**, *12*, 1444. https://doi.org/10.3390/biom12101444

Academic Editors: Jorge Joven, Fernández-Arroyo Salvador, Anna Hernández-Aguilera and Nuria Canela

Received: 31 August 2022
Accepted: 5 October 2022
Published: 9 October 2022

Publisher's Note: MDPI stays neutral with regard to jurisdictional claims in published maps and institutional affiliations.

Copyright: © 2022 by the authors. Licensee MDPI, Basel, Switzerland. This article is an open access article distributed under the terms and conditions of the Creative Commons Attribution (CC BY) license (https://creativecommons.org/licenses/by/4.0/).

The following supplementary materials are included:

- **Figure S1:** Comparison of prediction yields of immuno-inflammatory diseases and functions in the amplified and original small transcriptomics (miRNAs) datasets for the T1D high-risk (HR) and new-onset (NO)subject groups.

- **Tables S1A and S1B:** Complete lists of canonical pathways enriched for immuno-inflammatory processes in the original and amplified metabolomics datasets (A1, A2, and A3) of the (**S1A**) T1D high-risk (HR) and (**S1B**) new-onset (NO) subject groups, respectively.

- **Tables S2A and S2B:** Complete lists of canonical pathways enriched for immuno-inflammatory processes in the original and amplified proteomics datasets (A1, A2, and A3) of the (**S2A**) T1D high-risk (HR) and (**S2B**) new-onset (NO) subject groups, respectively.

- **Tables S3A and S3B:** Complete lists of immuno-inflammatory diseases & functions enriched in the integrated multi-omics datasets for (**S3A**) T1D high-risk (HR) and (**S3B**) new-onset (NO) subject groups, respectively.

- **Tables S4A and S4B:** Lists of consolidated biomarkers identified by *IPA*'s *Biomarker Prediction* module in the context of the selected diseases and functions in the proteomics and metabolomics amplified and original datasets for the (**S4A**) T1D high-risk (HR) and (**S4B**) new-onset (NO) subject groups, respectively.

Figure S1. Comparison of prediction yields of immuno-inflammatory diseases and functions in the amplified and original datasets of small transcriptomics (miRNAs). Analyses performed in *IPA* based on published literature available in its knowledge database as of the time of performing the analysis (https://go.qiagen.com/IPA-transcriptomics-whitepaper). Numbers of cononical pathways predicted in the amplified datasets (A1, A2, and A3) are shown as fold-change (increase or decrease) in comparison to the original data for the T1D high-risk (HR) and new-onset (NO) subject groups. None of the predictions were relevant to the T1D pathogenesis based on extensive search of the published literature as of the date of analyses.

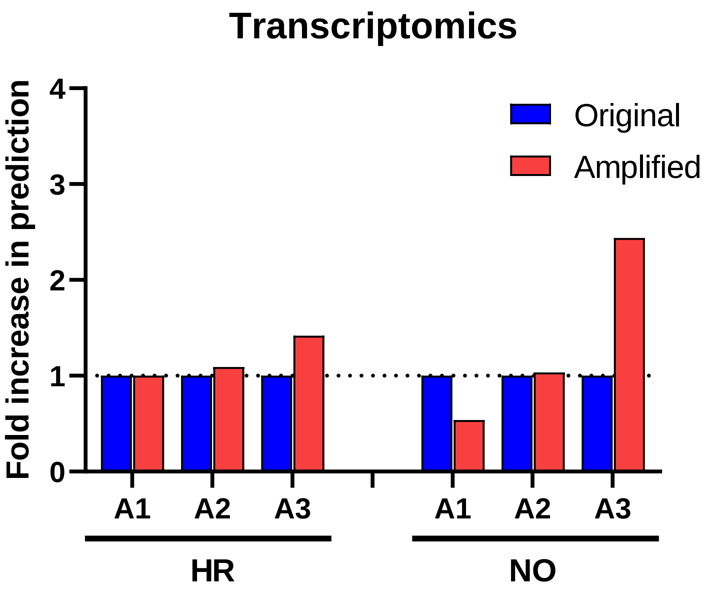

Table S1A: Complete list of canonical pathways identified in the T1D high-risk (HR) metabolomics datasets.

Canonical Pathways (HR)	Original	A1	A2	A3
Agranulocyte Adhesion and Diapedesis	15.2	28.4	14.2	14.2
tRNA Charging	6.53	16.6	7.92	7.9
Super-pathway of Citrulline Metabolism	6.28	13.7	7.5	7.49
Asparagine Biosynthesis I	6.05	8.9	5.53	5.52
Alanine Biosynthesis II	6.05	8.9	5.53	5.52
Alanine Degradation III	5.23	8.98	4.6	4.59
Arginine Degradation VI (Arginase 2 Pathway)	4.98	9.7	4.24	4.23
Citrulline Biosynthesis	4.68	11	5.05	5.03
Phenylalanine Degradation IV (Mammalian, via Side Chain)	4.32	6.15	5.35	5.33
Sirtuin Signaling Pathway	4	7.57	3.5	3.49
4-hydroxyproline Degradation I	4	7.23	3.5	3.49
Arginine Degradation I (Arginase Pathway)	3.94	6.55	3.55	3.55
4-hydroxyphenylpyruvate Biosynthesis	3.94	6.01	3.55	3.55
Aspartate Biosynthesis	3.94	6.25	3.55	3.55
Glycine Biosynthesis III	3.79	12.2	5.4	5.39
Arginine Biosynthesis IV	3.66	9.22	4.09	4.08
NAD biosynthesis II (from tryptophan)	3.44	6.5	2.95	2.94
Super-pathway of Serine and Glycine Biosynthesis I	3.29	6.15	2.81	2.8
Glycine Betaine Degradation	3.29	6.61	2.81	2.8
Proline Biosynthesis II (from Arginine)	3.29	8.6	3.41	3.4
Super-pathway of Methionine Degradation	3.25	7.98	4.39	4.38
Glutamate Degradation II	3.15	2.79	2.67	2.67
Endocannabinoid Neuronal Synapse Pathway	3.15	10.6	4.97	4.96
Urea Cycle	3.03	8.98	3.6	3.59
4-hydroxybenzoate Biosynthesis	3.03	6.61	2.55	2.54
5-aminoimidazole Ribonucleotide Biosynthesis I	3.02	5.48	2.65	2.64
4-aminobutyrate Degradation I	3.02	5.1	2.65	2.64
Aspartate Degradation II	3.02	5.22	2.65	2.64
Glutamate Degradation III (via 4-aminobutyrate)	3.02	2.82	2.65	2.64
Glutamate Receptor Signaling	3.02	5.34	2.65	2.64
L-cysteine Degradation I	3.02	5.63	2.65	2.64
L-cysteine Degradation III	2.83	7.76	3.74	3.74
Lipoate Biosynthesis and Incorporation II	2.83	5.48	2.46	2.45
β-alanine Degradation I	2.81	5.92	2.34	2.33
Leucine Degradation I	2.81	6.5	2.34	2.33
Uridine-5'-phosphate Biosynthesis	2.81	5.24	2.34	2.33
Valine Degradation I	2.69	3.84	2.14	2.13
Ferroptosis Signaling Pathway	2.69	10.4	3.77	3.76
Purine Nucleotides De Novo Biosynthesis II	2.66	5.63	2.3	2.29
(S)-reticuline Biosynthesis II	2.66	7.57	3.5	3.49
L-glutamine Biosynthesis II (tRNA-dependent)	2.66	5	2.3	2.29
Serine Biosynthesis	2.66	7.09	3.5	3.49
Tyrosine Degradation I	2.54	3.85	2.29	2.29
Anandamide Degradation	2.54	4.03	2.29	2.29
Asparagine Degradation I	2.54	4.03	2.29	2.29
Glutamine Degradation I	2.54	3.85	2.29	2.29
L-serine Degradation	2.52	5.1	2.16	2.15

Canonical Pathways (HR)	Original	A1	A2	A3
L-carnitine Biosynthesis	2.52	6.71	3.3	3.29
NAD Biosynthesis from 2-amino-3-carboxymuconate Semialdehyde	2.39	4.73	2.03	2.02
UDP-N-acetyl-D-glucosamine Biosynthesis II	2.37	4.48	1.92	1.91
Pyrimidine Ribonucleotides De Novo Biosynthesis	2.3	1.88	1.85	1.84
Insulin Secretion Signaling Pathway	2.27	3.33	1.82	1.91
Glutathione-mediated Detoxification	2.25	3.85	2	2
Glycine Biosynthesis I	2.25	4.03	2	2
Phosphatidylethanolamine Biosynthesis III	2.25	3.38	2	2
Pyruvate Fermentation to Lactate	2.25	3.71	2	2
Tyrosine Biosynthesis IV	2.17	5.24	2.81	2.8
Cysteine Biosynthesis III (mammalia)	2.17	4.65	1.82	1.81
Folate Polyglutamylation	2.1	2.12	1.67	1.66
Neurovascular Coupling Signaling Pathway	2.07	4.58	1.73	1.72
Lysine Degradation II	2.04	3.71	1.79	1.79
Glutamate Biosynthesis II	2.04	3.71	1.79	1.79
Glutamate Degradation X	2.04	3.59	1.79	1.79
Proline Degradation	2.04	3.59	1.79	1.79
S-adenosyl-L-methionine Biosynthesis	1.87	1.16	1.63	1.62
GABA Receptor Signaling	1.87	3.71	1.63	1.62
Glutamine Biosynthesis I	1.76	5.11	2.24	2.24
TCA Cycle II (Eukaryotic)	1.73	3.48	1.49	1.49
Glycine Degradation (Creatine Biosynthesis)	1.73	3.29	1.49	1.49
Phenylalanine Degradation I (Aerobic)	1.73	3.48	1.49	1.49
Threonine Degradation II	1.73	4.16	2.65	2.64
γ-linolenate Biosynthesis II (Animals)	1.7	4.06	1.37	1.36
Folate Transformations I	1.64	4.21	1.31	1.3
Lysine Degradation V	1.62	3.29	1.38	1.38
Glutathione Biosynthesis	1.62	2.39	1.38	1.38
Methionine Degradation I (to Homocysteine)	1.62	3.59	1.38	1.38
Morphine Biosynthesis	1.62	0.577	1.38	1.38
Synaptogenesis Signaling Pathway	1.52	1.25	1.28	1.28
Amyotrophic Lateral Sclerosis Signaling	1.52	3.08	1.28	1.28
dTMP De Novo Biosynthesis	1.52	1.74	1.28	1.28
Polyamine Regulation in Colon Cancer	1.52	0.883	1.28	1.28
Synaptic Long-Term Depression	1.52	0.943	1.28	1.28
Tumor Microenvironment Pathway	1.5	0.695	1.38	1.37
Basal Cell Carcinoma Signaling	1.5	1.15	1.38	1.37
Role of Lipids/Lipid Rafts in the Pathogenesis of Influenza	1.43	2.91	1.2	1.19
Adenine and Adenosine Salvage III	1.43	0.83	2.16	2.15
Hepatic Fibrosis Signaling Pathway	1.43	5	2.16	2.15
Molybdenum Cofactor Biosynthesis	1.43	3.15	1.2	1.19
Palmitate Biosynthesis I (Animals)	1.43	4.58	2.16	2.15
Phosphatidylethanolamine Biosynthesis II	1.43	3.08	1.2	1.19
Proline Biosynthesis I	1.43	4.82	2.16	2.15
Selenocysteine Biosynthesis II (Archaea and Eukaryotes)	1.43	2.96	1.2	1.19
Tryptophan Degradation to 2-amino-3-carboxymuconate Semialdehyde	1.43	2.24	1.2	1.19
Tryptophan Degradation X (Mammalian, via Tryptamine)	1.35	4.11	2.03	2.02

Canonical Pathways (HR)	Original	A1	A2	A3
Adenosine Nucleotides Degradation II	1.35	3.02	1.12	1.12
Catecholamine Biosynthesis	1.35	6.83	3.11	**3.1**
Citrulline-Nitric Oxide Cycle	1.35	3.15	1.12	1.12
L-DOPA Degradation	1.35	2.54	1.12	1.12
Leukotriene Biosynthesis	1.35	2.05	1.12	1.12
Pyrimidine Ribonucleotides Interconversion	1.28	2.61	1.06	1.05
Protein Kinase A Signaling	1.21	2.04	1.08	1.08
Glutamate Dependent Acid Resistance	1.21	2.04	1.08	1.08
MIF-mediated Glucocorticoid Regulation	1.21	2.1	1.82	1.81
Nitric Oxide Signaling in the Cardiovascular System	1.21	1.91	1.08	1.08
Putrescine Biosynthesis III	1.21	2.54	0.996	0.991
Dopamine-DARPP32 Feedback in cAMP Signaling	1.16	4.02	2.67	2.67
Stearate Biosynthesis I (Animals)	1.1	2.33	0.889	0.883
Neuroinflammation Signaling Pathway	1.05	1.78	0.842	0.839
Apelin Muscle Signaling Pathway	1.04	1.84	2.29	2.29
Role of MAPK Signaling in the Pathogenesis of Influenza	1.01	3.73	1.49	1.49
NAD Signaling Pathway	0.921	1.74	0.801	0.799
All-trans-decaprenyl Diphosphate Biosynthesis	0.921	1.67	0.801	0.799
IL-12 Signaling and Production in Macrophages	0.921	1.82	0.801	0.799
L-cysteine Degradation II	0.921	1.74	0.801	0.799
L-dopachrome Biosynthesis	0.921	4.03	2	2
Lipoate Salvage and Modification	0.921	1.56	0.801	0.799
Neuropathic Pain Signaling In Dorsal Horn Neurons	0.921	1.82	0.801	0.799
Sorbitol Degradation I	0.921	1.82	0.801	0.799
Thio-molybdenum Cofactor Biosynthesis	0.889	1.5	1.31	1.3
Gustation Pathway	0.863	4.48	1.04	1.03
Type II Diabetes Mellitus Signaling	0.83	1.56	0.712	0.71
2-ketoglutarate Dehydrogenase Complex	0.83	1.44	0.712	0.71
Acetyl-CoA Biosynthesis I (Pyruvate Dehydrogenase Complex)	0.83	1.56	0.712	0.71
D-myo-inositol (1,4,5)-trisphosphate Degradation	0.83	1.61	0.712	0.71
nNOS Signaling in Neurons	0.83	1.52	0.712	0.71
Trans, trans-farnesyl Diphosphate Biosynthesis	0.757	3.38	1.63	1.62
Antioxidant Action of Vitamin C	0.757	3.59	1.63	1.62
Cysteine Biosynthesis/Homocysteine Degradation	0.757	2.72	1.63	1.62
Fcγ Receptor-mediated Phagocytosis in Macrophages and Monocytes	0.757	1.61	0.642	0.64
Flavin Biosynthesis IV (Mammalian)	0.757	1.61	0.642	0.64
GDP-L-fucose Biosynthesis II (from L-fucose)	0.757	1.74	0.642	0.64
Glycine Biosynthesis II	0.757	1.41	0.642	0.64
Huntington's Disease Signaling	0.757	1.13	1.63	1.62
Insulin Receptor Signaling	0.757	1.56	0.642	0.64
Methionine Salvage II (Mammalian)	0.757	1.44	0.642	0.64
NAD Biosynthesis III	0.757	1.41	0.642	0.64
Oleate Biosynthesis II (Animals)	0.757	1.52	0.642	0.64
Oxidized GTP and dGTP Detoxification	0.757	1.32	0.642	0.64
Prostanoid Biosynthesis	0.757	1.61	0.642	0.64
Sulfate Activation for Sulfonation	0.757	1.35	1.63	1.62
Virus Entry via Endocytic Pathways	0.733	1.93	0.547	0.542
Tryptophan Degradation III (Eukaryotic)	0.697	1.61	0.583	0.582

Canonical Pathways (HR)	Original	A1	A2	A3
Acetyl-CoA Biosynthesis III (from Citrate)	0.697	3.38	1.49	1.49
Cancer Drug Resistance By Drug Efflux	0.697	1.61	0.583	0.582
Citrulline Degradation	0.697	1.56	0.583	0.582
Epoxysqualene Biosynthesis	0.697	3.15	1.49	1.49
GDP-mannose Biosynthesis	0.697	1.41	0.583	0.582
Glycerol Degradation I	0.697	1.41	0.583	0.582
Glycine Cleavage Complex	0.697	1.52	0.583	0.582
Pregnenolone Biosynthesis	0.697	1.93	1.49	1.49
tRNA Splicing	0.697	3.38	1.49	1.49
Uracil Degradation II (Reductive)	0.697	1.56	0.583	0.582
Xanthine and Xanthosine Salvage	0.682	2.17	0.5	0.496
Cholesterol Biosynthesis I	0.682	2.17	0.5	0.496
Cholesterol Biosynthesis II (via 24,25-dihydrolanosterol)	0.682	2.17	0.5	0.496
Cholesterol Biosynthesis III (via Desmosterol)	0.646	1.48	0.535	0.533
Glioma Signaling	0.646	1.56	0.535	0.533
Synaptic Long-Term Potentiation	0.646	1.41	0.535	0.533
Tetrapyrrole Biosynthesis II	0.6	3.22	1.28	1.28
Ketolysis	0.6	2.26	1.28	1.28
NAD Salvage Pathway II	0.6	1.48	0.492	0.491
D-myo-inositol (1,4,5)-Trisphosphate Biosynthesis	0.561	2.09	1.2	1.19
CDP-diacylglycerol Biosynthesis I	0.526	1.37	0.423	0.42
Coenzyme A Biosynthesis	0.526	1.41	0.423	0.42
Dolichol and Dolichyl Phosphate Biosynthesis	0.526	2.28	1.12	1.12
CMP-N-acetylneuraminate Biosynthesis I (Eukaryotes)	0.495	1.73	1.06	1.05
3-phosphoinositide Biosynthesis	0.467	2	0.996	0.991
Gap Junction Signaling	0.467	2.96	0.996	0.991
UDP-N-acetyl-D-galactosamine Biosynthesis II	0.396	2.11	0.842	0.839
Mitochondrial Dysfunction	0.376	1.31	0.799	0.796
Super-pathway of Geranylgeranyldiphosphate Biosynthesis I (via Mevalonate)	0.308	2.03	0.656	0.652
Gluconeogenesis I	0.294	2.26	0.627	0.622
Colanic Acid Building Blocks Biosynthesis	0.264	1.54		
2-oxobutanoate Degradation I		1.82	0.801	0.799
Cellular Effects of Sildenafil (Viagra)		1.37	0.583	0.582
Ceramide Degradation		2.04	1.08	1.08
Leptin Signaling in Obesity		1.44	0.535	0.533
Protein Ubiquitination Pathway		1.61	0.801	0.799
Super-pathway of Melatonin Degradation		1.82	0.801	0.799
Thiamin Salvage III	15.2	28.4	14.2	14.2

Footnote: Shown values = -log(p) for each predicted canonical pathway. Only significant predictions with -log(p) > 1.3 (i.e., $p < 0.05$) were shown; however, when significance was reached in at least one of the datasets (original or amplified), non-significant predictions with -log(p) values < 1.3 were shown for comparison. Blank means no prediction.

Table S1B: Complete list of canonical pathways identified in the T1D new-onset (NO) metabolomics datasets.

Canonical Pathways (NO)	Original	A1	A2	A3
tRNA Charging	16.3	28.4	14.2	14.2
Asparagine Biosynthesis I	6.57	13.7	7.5	7.49
Super-pathway of Citrulline Metabolism	5.7	16.6	7.92	7.9
Alanine Biosynthesis II	4.1	8.9	5.53	5.52
Alanine Degradation III	4.1	8.9	5.53	5.52
Glycine Biosynthesis III	4.1	6.25	3.55	3.55
Citrulline Biosynthesis	4.06	9.7	4.24	4.23
Arginine Degradation VI (Arginase 2 Pathway)	4.01	8.98	4.6	4.59
NAD biosynthesis II (from tryptophan)	3.94	9.22	4.09	4.08
Phenylalanine Degradation IV (Mammalian, via Side Chain)	3.82	11	5.05	5.03
Glycine Betaine Degradation	3.51	6.15	2.81	2.8
Endocannabinoid Neuronal Synapse Pathway	3.37	2.79	2.67	2.67
Urea Cycle	3.37	10.6	4.97	4.96
5-aminoimidazole Ribonucleotide Biosynthesis I	3.24	6.61	2.55	2.54
Glutamate Receptor Signaling	3.19	2.82	2.65	2.64
Uridine-5'-phosphate Biosynthesis	3.02	6.5	2.34	2.33
Lipoate Biosynthesis and Incorporation II	2.99	7.76	3.74	3.74
Purine Nucleotides De Novo Biosynthesis II	2.95	10.4	3.77	3.76
Arginine Biosynthesis IV	2.92	12.2	5.4	5.39
4-hydroxyproline Degradation I	2.82	7.57	3.5	3.49
Arginine Degradation I (Arginase Pathway)	2.82	7.23	3.5	3.49
L-glutamine Biosynthesis II (tRNA-dependent)	2.82	7.57	3.5	3.49
Sirtuin Signaling Pathway	2.75	6.15	5.35	5.33
Super-pathway of Methionine Degradation	2.69	8.6	3.41	3.4
NAD Biosynthesis from 2-amino-3-carboxymuconate Semialdehyde	2.68	6.71	3.3	3.29
Anandamide Degradation	2.65	3.85	2.29	2.29
Asparagine Degradation I	2.65	4.03	2.29	2.29
Glutamine Degradation I	2.65	4.03	2.29	2.29
L-serine Degradation	2.65	3.85	2.29	2.29
Pyrimidine Ribonucleotides De Novo Biosynthesis	2.58	4.48	1.92	1.91
UDP-N-acetyl-D-glucosamine Biosynthesis II	2.55	4.73	2.03	2.02
Glutathione-mediated Detoxification	2.43	3.33	1.82	1.91
Super-pathway of Serine and Glycine Biosynthesis I	2.43	6.5	2.95	2.94
4-hydroxyphenylpyruvate Biosynthesis	2.36	6.55	3.55	3.55
Aspartate Biosynthesis	2.36	6.01	3.55	3.55
Glycine Biosynthesis I	2.36	3.85	2	2
Phosphatidylethanolamine Biosynthesis III	2.36	4.03	2	2
Pyruvate Fermentation to Lactate	2.36	3.38	2	2
Tyrosine Biosynthesis IV	2.36	3.71	2	2
Cysteine Biosynthesis III (mammalia)	2.33	5.24	2.81	2.8
Folate Polyglutamylation	2.33	4.65	1.82	1.81
Proline Biosynthesis II (from Arginine)	2.33	6.61	2.81	2.8
Neurovascular Coupling Signaling Pathway	2.3	2.12	1.67	1.66
Proline Degradation	2.15	3.59	1.79	1.79
S-adenosyl-L-methionine Biosynthesis	2.15	3.59	1.79	1.79
4-hydroxybenzoate Biosynthesis	2.14	8.98	3.6	3.59

Canonical Pathways (NO)	Original	A1	A2	A3
Ferroptosis Signaling Pathway	2.07	3.84	2.14	2.13
Glutamate Degradation II	1.98	7.98	4.39	4.38
Glutamine Biosynthesis I	1.98	3.71	1.63	1.62
Leucine Degradation I	1.98	5.92	2.34	2.33
Valine Degradation I	1.98	5.24	2.34	2.33
Folate Transformations I	1.85	4.06	1.37	1.36
Aspartate Degradation II	1.84	5.1	2.65	2.64
Glycine Degradation (Creatine Biosynthesis)	1.84	3.48	1.49	1.49
L-cysteine Degradation I	1.84	5.34	2.65	2.64
L-cysteine Degradation III	1.84	5.63	2.65	2.64
Phenylalanine Degradation I (Aerobic)	1.84	3.29	1.49	1.49
Threonine Degradation II	1.84	3.48	1.49	1.49
γ-linolenate Biosynthesis II (Animals)	1.84	4.16	2.65	2.64
Glutathione Biosynthesis	1.72	3.29	1.38	1.38
Methionine Degradation I (to Homocysteine)	1.72	2.39	1.38	1.38
Synaptogenesis Signaling Pathway	1.72	0.577	1.38	1.38
(S)-reticuline Biosynthesis II	1.62	5.63	2.3	2.29
Amyotrophic Lateral Sclerosis Signaling	1.62	1.25	1.28	1.28
dTMP De Novo Biosynthesis	1.62	3.08	1.28	1.28
Insulin Secretion Signaling Pathway	1.62	1.88	1.85	1.84
Polyamine Regulation in Colon Cancer	1.62	1.74	1.28	1.28
Serine Biosynthesis	1.62	5	2.3	2.29
Synaptic Long-Term Depression	1.62	0.883	1.28	1.28
Tumor Microenvironment Pathway	1.62	0.943	1.28	1.28
Tyrosine Degradation I	1.62	7.09	3.5	3.49
Basal Cell Carcinoma Signaling	1.56	0.695	1.38	1.37
Role of Lipids/Lipid Rafts in the Pathogenesis of Influenza	1.56	1.15	1.38	1.37
Adenine and Adenosine Salvage III	1.53	2.91	1.2	1.19
Molybdenum Cofactor Biosynthesis	1.53	5	2.16	2.15
Phosphatidylethanolamine Biosynthesis II	1.53	4.58	2.16	2.15
Proline Biosynthesis I	1.53	3.08	1.2	1.19
Selenocysteine Biosynthesis II (Archaea and Eukaryotes)	1.53	4.82	2.16	2.15
Tryptophan Degradation to 2-amino-3-carboxymuconate Semialdehyde	1.53	2.96	1.2	1.19
Tryptophan Degradation X (Mammalian, via Tryptamine)	1.53	2.24	1.2	1.19
Adenosine Nucleotides Degradation II	1.45	4.11	2.03	2.02
Citrulline-Nitric Oxide Cycle	1.45	6.83	3.11	3.1
Leukotriene Biosynthesis	1.45	2.54	1.12	1.12
Pyrimidine Ribonucleotides Interconversion	1.45	2.05	1.12	1.12
Glucose and Glucose-1-phosphate Degradation	1.38	2.61	1.06	1.05
Protein Kinase A Signaling	1.38	0.428	1.06	1.05
Circadian Rhythm Signaling	1.32	0.668	0.996	0.991
Nitric Oxide Signaling in the Cardiovascular System	1.32	2.1	1.82	1.81
γ-glutamyl Cycle	1.32	2.54	0.996	0.991
Alanine Biosynthesis III	1.26	2.04	1.08	1.08
Glutamate Dependent Acid Resistance	1.26	1.91	1.08	1.08
Glutamate Removal from Folates	1.26	2.04	1.08	1.08
Lysine Degradation II	1.26	4.58	1.73	1.72
Putrescine Biosynthesis III	1.26	1.91	1.08	1.08

Canonical Pathways (NO)	Original	A1	A2	A3
Stearate Biosynthesis I (Animals)	1.26	4.02	2.67	2.67
PFKFB4 Signaling Pathway	1.15	1.78	0.842	0.839
Purine Nucleotides Degradation II (Aerobic)	1.1	3.73	1.49	1.49
FAT10 Signaling Pathway	1.09	1.84	2.29	2.29
Bile Acid Biosynthesis, Neutral Pathway	0.991	4.48	1.04	1.03
Gustation Pathway	0.983	1.5	1.31	1.3
Lysine Degradation V	0.983	4.21	1.31	1.3
All-trans-decaprenyl Diphosphate Biosynthesis	0.975	1.74	0.801	0.799
Geranylgeranyldiphosphate Biosynthesis	0.975	1.67	0.801	0.799
L-cysteine Degradation II	0.975	1.82	0.801	0.799
L-dopachrome Biosynthesis	0.975	1.74	0.801	0.799
Lipoate Salvage and Modification	0.975	4.03	2	2
Myo-inositol Biosynthesis	0.975	1.56	0.801	0.799
Sorbitol Degradation I	0.975	1.82	0.801	0.799
Thio-molybdenum Cofactor Biosynthesis	0.975	1.82	0.801	0.799
Acetyl-CoA Biosynthesis I (Pyruvate Dehydrogenase Complex)	0.883	1.44	0.712	0.71
Branched-chain α-keto acid Dehydrogenase Complex	0.883	1.56	0.712	0.71
Glutamate Biosynthesis II	0.883	3.71	1.79	1.79
Glutamate Degradation X	0.883	3.71	1.79	1.79
Methylglyoxal Degradation I	0.883	1.61	0.712	0.71
Trans, trans-farnesyl Diphosphate Biosynthesis	0.883	1.52	0.712	0.71
Tryptophan Degradation III (Eukaryotic)	0.821	1.93	0.547	0.542
Acetate Conversion to Acetyl-CoA	0.81	3.38	1.63	1.62
Cysteine Biosynthesis/Homocysteine Degradation	0.81	3.59	1.63	1.62
Fatty Acid Activation	0.81	2.72	1.63	1.62
Flavin Biosynthesis IV (Mammalian)	0.81	1.61	0.642	0.64
GABA Receptor Signaling	0.81	1.16	1.63	1.62
GDP-L-fucose Biosynthesis II (from L-fucose)	0.81	1.61	0.642	0.64
Glycine Biosynthesis II	0.81	1.74	0.642	0.64
Glycogen Biosynthesis II (from UDP-D-Glucose)	0.81	1.41	0.642	0.64
Insulin Receptor Signaling	0.81	1.13	1.63	1.62
Methionine Salvage II (Mammalian)	0.81	1.56	0.642	0.64
NAD Biosynthesis III	0.81	1.44	0.642	0.64
NAD Salvage Pathway III	0.81	1.41	0.642	0.64
Oxidized GTP and dGTP Detoxification	0.81	1.52	0.642	0.64
Prostanoid Biosynthesis	0.81	1.32	0.642	0.64
Sulfate Activation for Sulfonation	0.81	1.61	0.642	0.64
Sumoylation Pathway	0.81	1.35	1.63	1.62
Cholesterol Biosynthesis I	0.767	2.17	0.5	0.496
Cholesterol Biosynthesis II (via 24,25-dihydrolanosterol)	0.767	2.17	0.5	0.496
Cholesterol Biosynthesis III (via Desmosterol)	0.767	2.17	0.5	0.496
4-aminobutyrate Degradation I	0.747	5.48	2.65	2.64
Acetyl-CoA Biosynthesis III (from Citrate)	0.747	1.61	0.583	0.582
Adenine and Adenosine Salvage I	0.747	3.38	1.49	1.49
Citrulline Degradation	0.747	1.61	0.583	0.582
Epoxysqualene Biosynthesis	0.747	1.56	0.583	0.582
GDP-mannose Biosynthesis	0.747	3.15	1.49	1.49
Glutamate Degradation III (via 4-aminobutyrate)	0.747	5.22	2.65	2.64
Glycerol Degradation I	0.747	1.41	0.583	0.582

Canonical Pathways (NO)	Original	A1	A2	A3
Glycine Cleavage Complex	0.747	1.41	0.583	0.582
Guanine and Guanosine Salvage I	0.747	1.52	0.583	0.582
tRNA Splicing	0.747	1.93	1.49	1.49
Xanthine and Xanthosine Salvage	0.747	1.56	0.583	0.582
Methylglyoxal Degradation VI	0.695	1.56	0.535	0.533
Tetrapyrrole Biosynthesis II	0.695	1.41	0.535	0.533
β-alanine Degradation I	0.695	5.48	2.46	2.45
Biotin-carboxyl Carrier Protein Assembly	0.65	3.22	1.28	1.28
NAD Salvage Pathway II	0.65	2.26	1.28	1.28
S-methyl-5-thio-α-D-ribose 1-phosphate Degradation	0.65	1.48	0.492	0.491
Ethanol Degradation II	0.609	2.09	1.2	1.19
Hepatic Fibrosis Signaling Pathway	0.609	0.83	2.16	2.15
L-carnitine Biosynthesis	0.609	5.1	2.16	2.15
Palmitate Biosynthesis I (Animals)	0.609	3.15	1.2	1.19
Catecholamine Biosynthesis	0.573	3.02	1.12	1.12
Coenzyme A Biosynthesis	0.573	1.37	0.423	0.42
Dolichol and Dolichyl Phosphate Biosynthesis	0.573	1.41	0.423	0.42
Ethanol Degradation IV	0.573	2.28	1.12	1.12
L-DOPA Degradation	0.573	3.15	1.12	1.12
Oxidative Ethanol Degradation III	0.542	1.73	1.06	1.05
Fatty Acid β-oxidation I	0.513	2	0.996	0.991
Inosine-5'-phosphate Biosynthesis II	0.513	2.96	0.996	0.991
Isoleucine Degradation I	0.462	2.33	0.889	0.883
Glycolysis I	0.44	2.11	0.842	0.839
Salvage Pathways of Pyrimidine Ribonucleotides	0.419	1.31	0.799	0.796
TCA Cycle II (Eukaryotic)	0.399	5.11	2.24	2.24
Gluconeogenesis I	0.349	2.03	0.656	0.652
Colanic Acid Building Blocks Biosynthesis	0.334	2.26	0.627	0.622
Super-pathway of Cholesterol Biosynthesis	0.324	1.54		
2-ketoglutarate Dehydrogenase Complex		1.56	0.712	0.71
Ascorbate Recycling (Cytosolic)		1.48	0.535	0.533
Ceramide Degradation		1.37	0.583	0.582
D-mannose Degradation		2.04	1.08	1.08
Melatonin Degradation II		1.44	0.535	0.533
Morphine Biosynthesis		3.59	1.38	1.38
PRPP Biosynthesis I		1.61	0.801	0.799
Thiamin Salvage III		1.82	0.801	0.799
Uracil Degradation II (Reductive)		3.38	1.49	1.49

Footnote: Shown values = -log(p) for each predicted canonical pathway. Only significant predictions with -log(p) > 1.3 (i.e., $p < 0.05$) were shown; however, when significance was reached in at least one of the datasets (original or amplified), non-significant predictions with -log(p) values < 1.3 were shown for comparison. Blank means no prediction.

Table S2A: Complete list of canonical pathways identified in the T1D high-risk (HR) proteomics datasets.

Canonical Pathways (HR)	Original	A1	A2	A3
Agranulocyte Adhesion and Diapedesis	5.44	5.38	5.39	2.27
Inhibition of Matrix Metalloproteases	5.07	5.03	5.04	1.19
Leukocyte Extravasation Signaling	4.95	4.04	4.05	5.4
Adenine and Adenosine Salvage III	4.9	4.88	4.88	0.81
Glutathione Redox Reactions I	4.34	4.31	4.31	0.848
Granulocyte Adhesion and Diapedesis	4.16	4.1	4.11	1.08
Heparan Sulfate Biosynthesis (Late Stages)	3.58	3.54	3.5	0.539
LXR/RXR Activation	3.57	3.53	3.54	11.9
Phenylethylamine Degradation I	3.52	3.5	3.5	1.04
Tumor Microenvironment Pathway	3.49	3.45	3.45	3.99
Heparan Sulfate Biosynthesis	3.4	3.37	3.33	0.475
Iron homeostasis signaling pathway	3.29	3.25	3.25	2.74
Hepatic Fibrosis / Hepatic Stellate Cell Activation	3.28	3.24	4.03	5.37
Pentose Phosphate Pathway (Non-oxidative Branch)	3.12	3.11	3.11	
Purine Ribonucleosides Degradation to Ribose-1-phosphate	2.85	2.84	2.84	0.757
Glioma Invasiveness Signaling	2.72	2.69	2.7	1.53
IL-15 Production	2.71	2.67	3.54	7.42
FXR/RXR Activation	2.66	2.63	2.63	9.81
Sperm Motility	2.6	2.55	3.23	6.05
Atherosclerosis Signaling	2.59	2.55	2.56	7.83
Pulmonary Fibrosis Idiopathic Signaling Pathway	2.59	2.55	3.17	4.9
Pentose Phosphate Pathway	2.57	2.55	2.55	
Pulmonary Healing Signaling Pathway	2.49	2.45	3.18	2.08
HIPPO signaling	2.48	1.61	1.61	0.842
HIF1α Signaling	2.4	2.36	2.37	3.32
IL-8 Signaling	2.38	2.34	2.35	6.8
Apelin Adipocyte Signaling Pathway	2.37	2.35	2.35	0.434
Adenosine Nucleotides Degradation II	2.24	2.22	2.23	0.496
Phagosome Maturation	2.23	2.94	2.2	1.44
HOTAIR Regulatory Pathway	2.18	2.15	2.16	1.79
IGF-1 Signaling	2.17	2.14	2.15	1.92
FAT10 Signaling Pathway	2.12	2.1	2.1	0.83
Primary Immunodeficiency Signaling	2.12	3.12	2.1	2.68
Triacylglycerol Degradation	2.1	2.07	2.04	
Purine Nucleotides Degradation II (Aerobic)	2.09	2.08	2.08	0.435
Bladder Cancer Signaling	2	1.98	1.98	1.68
Neuroprotective Role of THOP1 in Alzheimer's Disease	1.95	2.72	2.72	1.61
Phospholipases	1.9	1.88	1.89	1.12
Colorectal Cancer Metastasis Signaling	1.86	1.83	1.83	3.08
Asparagine Degradation I	1.84	1.84	1.84	1.33

Canonical Pathways (HR)	Original	A1	A2	A3
ID1 Signaling Pathway	1.83	1.8	3.16	3.49
Huntington's Disease Signaling	1.77	1.74	1.75	2.47
Guanine and Guanosine Salvage I	1.67	1.66	1.66	
BAG2 Signaling Pathway	1.64	1.62	1.63	0.492
Role of Osteoblasts, Osteoclasts and Chondrocytes in Rheumatoid Arthritis	1.64	1.61	1.09	1.73
Ovarian Cancer Signaling	1.56	1.54	1.54	1.45
Crosstalk between Dendritic Cells and Natural Killer Cells	1.55	0.845	0.312	2.84
Estrogen Receptor Signaling	1.55	1.51	1.51	2.71
Heme Degradation	1.55	1.54	1.54	1.04
PRPP Biosynthesis I	1.55	1.54	1.54	1.04
Intrinsic Prothrombin Activation Pathway	1.44	0.577	0.577	2.51
LPS/IL-1 Mediated Inhibition of RXR Function	1.43	1.41	1.41	1.09
Erythropoietin Signaling Pathway	1.41	1.38	1.39	1.59
B Cell Development	1.4	2.39	1.39	0.551
Amyotrophic Lateral Sclerosis Signaling	1.3	1.99	1.27	0.857
Clathrin-mediated Endocytosis Signaling	1.29	1.86	1.27	13.7
Regulation of the Epithelial-Mesenchymal Transition Pathway	1.28	1.26	1.84	2.15
RHOA Signaling	1.22	1.2	1.2	7.37
Axonal Guidance Signaling	1.13	1.1	1.1	9.82
Protein Kinase A Signaling	1.12	1.09	0.457	3.07
MSP-RON Signaling In Cancer Cells Pathway	1.1	1.08	1.08	3.88
Semaphorin Neuronal Repulsive Signaling Pathway	1.02	1	1.01	2.48
Maturity Onset Diabetes of Young (MODY) Signaling	0.967	0.951	0.947	3.28
Gαq Signaling	0.907	0.889	0.893	3.1
Protein Ubiquitination Pathway	0.863	1.28	1.29	2.2
Fcγ Receptor-mediated Phagocytosis in Macrophages and Monocytes	0.833	0.821	0.824	9.16
Acute Phase Response Signaling	0.827	1.33	0.815	6.32
Tumoricidal Function of Hepatic Natural Killer Cells	0.799	0.79	0.793	6
Endothelin-1 Signaling	0.793	0.78	0.783	2.19
Regulation of the Epithelial Mesenchymal Transition by Growth Factors Pathway	0.793	0.78	1.28	2.65
Role of Tissue Factor in Cancer	0.693	0.682	0.684	3.99
MSP-RON Signaling In Macrophages Pathway	0.676	0.666	0.668	1.63
Inhibition of Angiogenesis by TSP1	0.662	0.656	0.658	2.08
Coagulation System	0.652			2.04
Pancreatic Adenocarcinoma Signaling	0.64	0.629	0.631	1.98
Phagosome Formation	0.638	0.87	0.873	4.6
Complement System	0.631	0.623	0.625	5.69
Glutathione-mediated Detoxification	0.631	0.623	0.625	1.95
STAT3 Pathway	0.597	0.585	1.12	1.81

Canonical Pathways (HR)	Original	A1	A2	A3
IL-12 Signaling and Production in Macrophages	0.592	0.582	0.583	4.65
RAC Signaling	0.583	0.572	0.573	1.33
Apelin Endothelial Signaling Pathway	0.57	0.559	0.561	1.71
PI3K Signaling in B Lymphocytes	0.551			2.65
Aryl Hydrocarbon Receptor Signaling	0.499	0.489	0.491	1.44
Lymphotoxin β Receptor Signaling	0.484	0.479	0.479	1.38
MSP-RON Signaling Pathway	0.466	0.46	0.461	2.6
IL-2 Signaling	0.449	0.442	0.442	1.83
Synaptogenesis Signaling Pathway	0.399	0.693	0.389	5.61
Production of Nitric Oxide and Reactive Oxygen Species in Macrophages	0.398	0.389	0.39	2.67
GNRH Signaling	0.396	0.386	0.388	1.39
Ephrin B Signaling	0.391	0.386	0.387	6.92
Adrenomedullin signaling pathway	0.377	0.369	0.37	2.08
Natural Killer Cell Signaling	0.377	0.369		2.08
Role of Macrophages, Fibroblasts and Endothelial Cells in Rheumatoid Arthritis	0.377	0.366		5.37
Macropinocytosis Signaling	0.374	0.369	0.37	5.76
Thyroid Cancer Signaling	0.362	0.356	0.357	1.95
Chemokine Signaling	0.358	0.352	0.353	5.53
Estrogen-Dependent Breast Cancer Signaling	0.354	0.348	0.349	1.9
Role of MAPK Signaling in the Pathogenesis of Influenza	0.35	0.344	0.345	1.87
VEGF Family Ligand-Receptor Interactions	0.342	0.336	0.337	1.3
RHOGDI Signaling	0.338	0.33	0.332	3.68
NRF2-mediated Oxidative Stress Response	0.292	0.285	0.602	2.79
VEGF Signaling	0.292	0.287	0.287	4.63
Sumoylation Pathway	0.28	0.275	0.276	1.44
Paxillin Signaling	0.266	0.261	0.262	7.39
α-Adrenergic Signaling	0.266	0.261	0.262	1.36
Wound Healing Signaling Pathway	0.265	0.258	0.259	2.96
Cardiac Hypertrophy Signaling	0.252	0.245	0.244	2.83
IL-13 Signaling Pathway	0.246	0.241	0.242	2.74
PAK Signaling	0.241	0.237	0.237	5.32
Signaling by Rho Family GTPases	0.24	0.232	0.234	4.6
Cholecystokinin/Gastrin-mediated Signaling	0.239	0.234	0.235	6.03
Sphingosine-1-phosphate Signaling	0.237	0.232	0.233	3.23
Renin-Angiotensin Signaling	0.232	0.227	0.228	1.58
Glioma Signaling	0.228	0.223	0.224	4.39
G Beta Gamma Signaling	0.217	0.213	0.213	1.92
fMLP Signaling in Neutrophils	0.213	0.208	0.209	1.43
Ferroptosis Signaling Pathway	0.211	0.206	0.602	2.36
Gα12/13 Signaling	0.209	0.205	0.206	4.09

Canonical Pathways (HR)	Original	A1	A2	A3
CCR3 Signaling in Eosinophils	0.206	0.201	0.201	4.03
SNARE Signaling Pathway	0.203	0.199	0.199	2.28
14-3-3-mediated Signaling				1.49
Actin Cytoskeleton Signaling				11.1
Actin Nucleation by ARP-WASP Complex				5.68
Adenine and Adenosine Salvage VI				1.62
Agrin Interactions at Neuromuscular Junction				6.12
Angiopoietin Signaling				4.86
Apoptosis Signaling				2.47
Autophagy				1.48
Caveolar-mediated Endocytosis Signaling		0.373		7.66
Cellular Effects of Sildenafil (Viagra)				2.52
Chronic Myeloid Leukemia Signaling				1.37
CLEAR Signaling Pathway			0.462	1.39
CNTF Signaling				1.95
Colanic Acid Building Blocks Biosynthesis				1.37
Coronavirus Replication Pathway				4.07
CXCR4 Signaling				5.53
Death Receptor Signaling				4.76
EGF Signaling				2.72
Endocannabinoid Cancer Inhibition Pathway				1.61
Endometrial Cancer Signaling				1.86
Ephrin A Signaling				1.6
Ephrin Receptor Signaling				10.7
Epithelial Adherens Junction Signaling				6.66
ERBB Signaling				3.4
ERK/MAPK Signaling				1.84
ERK5 Signaling				2.73
FcγRIIB Signaling in B Lymphocytes		0.333	0.333	2.39
Gap Junction Signaling				1.69
GDNF Family Ligand-Receptor Interactions				4.11
GDP-mannose Biosynthesis				2.1
Germ Cell-Sertoli Cell Junction Signaling				3.08
Glioblastoma Multiforme Signaling				4.82
Gluconeogenesis I				2.51
Glutamate Removal from Folates				1.62
Glycolysis I				2.45
GM-CSF Signaling				1.6
GP6 Signaling Pathway				4.29
Granzyme B Signaling				2.23
Hepatic Fibrosis Signaling Pathway				3.29
HER-2 Signaling in Breast Cancer				2.08

Canonical Pathways (HR)	Original	A1	A2	A3
IL-6 Signaling				1.94
IL-7 Signaling Pathway		0.361	0.361	1.97
ILK Signaling				5.2
Induction of Apoptosis by HIV1				2.35
Integrin Signaling				13.5
Leukotriene Biosynthesis				1.32
Melanocyte Development and Pigmentation Signaling				1.53
Molecular Mechanisms of Cancer				2.6
MYC Mediated Apoptosis Signaling				2.19
Neuregulin Signaling				2.71
Neurotrophin/TRK Signaling				1.42
NF-κB Activation by Viruses				1.42
NGF Signaling				2.64
Non-Small Cell Lung Cancer Signaling				2.15
Oncostatin M Signaling		0.567		1.73
Opioid Signaling Pathway				1.44
Oxytocin Signaling Pathway				4.32
p70S6K Signaling		0.206	0.207	2.91
PDGF Signaling				3
PEDF Signaling				3.06
PPAR Signaling				1.85
PPARα/RXRα Activation				1.36
Prostate Cancer Signaling				1.72
PTEN Signaling			0.524	2.01
Pyridoxal 5'-phosphate Salvage Pathway				1.69
Regulation of Actin-based Motility by Rho				3.34
Regulation of Cellular Mechanics by Calpain Protease				4.3
Remodeling of Epithelial Adherens Junctions		0.406		5.35
Renal Cell Carcinoma Signaling				4.71
Role of PKR in Interferon Induction and Antiviral Response				2.28
Semaphorin Signaling in Neurons				3.19
Sucrose Degradation V (Mammalian)				1.57
Telomerase Signaling				1.85
Thrombin Signaling				4.53
Tight Junction Signaling				2.44
TNFR1 Signaling				2.85
TWEAK Signaling				1.95
UVC-Induced MAPK Signaling				1.49
Virus Entry via Endocytic Pathways				4.43

Footnote: Shown values = -log(p) for each predicted canonical pathway. Only significant predictions with -log(p) > 1.3 (i.e., $p < 0.05$) were shown; however, when significance was reached in at least one of the datasets (original or amplified), non-significant predictions with -log(p) values < 1.3 were shown for comparison. Blank means no prediction.

Table S2B: Complete list of canonical pathways identified in the new-onset (NO) proteomics datasets.

Canonical Pathways (NO)	Original	A1	A2	A3
LXR/RXR Activation	13.6	13.6	13.6	9.91
IL-15 Production	12.5	12.5	10.5	5.06
FXR/RXR Activation	10.3	10.3	11.3	7.2
Hepatic Fibrosis / Hepatic Stellate Cell Activation	9.83	8.97	9.89	7.28
Atherosclerosis Signaling	9.06	9.05	9.11	6.2
Sperm Motility	8.49	7.74	6.41	5.96
Complement System	8.05	8.04	8.08	4.61
Clathrin-mediated Endocytosis Signaling	7.42	6.64	6.7	9.52
Tumor Microenvironment Pathway	5.6	4.88	5.63	5.14
Acute Phase Response Signaling	5.43	5.41	5.46	9.89
Inhibition of Matrix Metalloproteases	5.42	5.42	4.34	2.63
Primary Immunodeficiency Signaling	5.34	4.35	5.36	5.16
Maturity Onset Diabetes of Young (MODY) Signaling	5.15	5.14	4.25	1.95
Agranulocyte Adhesion and Diapedesis	4.7	4.69	4.74	2.67
Granulocyte Adhesion and Diapedesis	4.64	4.63	4.67	1.06
Axonal Guidance Signaling	4.63	3.71	3.76	12
Iron homeostasis signaling pathway	4.57	3.85	3.89	2.7
Leukocyte Extravasation Signaling	4.54	4.53	3.94	4.15
Neuroprotective Role of THOP1 in Alzheimer's Disease	4.4	4.39	4.42	0.788
IL-12 Signaling and Production in Macrophages	3.94	3.93	3.96	2.24
Adenine and Adenosine Salvage III	3.79	3.79	3.8	0.804
Pulmonary Fibrosis Idiopathic Signaling Pathway	3.79	3.77	2.85	2.6
Role of Macrophages, Fibroblasts and Endothelial Cells in Rheumatoid Arthritis	3.75	3.74	3.79	7.49
Coagulation System	3.53	3.52	3.54	0.241
CLEAR Signaling Pathway	3.39	2.88	3.95	3.23
LPS/IL-1 Mediated Inhibition of RXR Function	3.35	2.83	3.38	2.07
STAT3 Pathway	3.28	2.65	3.31	1.78
Intrinsic Prothrombin Activation Pathway	3.15	3.15	3.17	
Phenylethylamine Degradation I	2.77	2.77	2.78	2.47
Macropinocytosis Signaling	2.73	2.72	2.74	8.49
Leukotriene Biosynthesis	2.72	2.71	2.73	3.44
Heparan Sulfate Biosynthesis (Late Stages)	2.64	3.39	2.6	1.38
Pulmonary Healing Signaling Pathway	2.64	2.64	2.67	2.48
Extrinsic Prothrombin Activation Pathway	2.63	2.63	2.64	0.491
ID1 Signaling Pathway	2.63	2.62	2.65	
FAT10 Signaling Pathway	2.58	2.58	2.6	4.26
Heparan Sulfate Biosynthesis	2.45	3.17	2.42	1.25
Role of Osteoblasts, Osteoclasts and Chondrocytes in Rheumatoid Arthritis	2.28	1.82	2.3	1.03
Production of Nitric Oxide and Reactive Oxygen Species in Macrophages	2.25	2.24	2.27	2.63
Regulation of the Epithelial Mesenchymal Transition by Growth Factors Pathway	2.24	2.23	1.78	4.17
Regulation of the Epithelial-Mesenchymal Transition Pathway	2.19	2.19	2.21	3.03
HOTAIR Regulatory Pathway	2.16	2.15	2.73	1.76
Purine Ribonucleosides Degradation to Ribose-1-phosphate	2.12	2.12	2.13	0.752

Canonical Pathways (NO)	Original	A1	A2	A3
Ephrin Receptor Signaling	2.08	1.63	1.65	8.35
Synaptogenesis Signaling Pathway	2.07	2.06	1.71	5.52
HIF1α Signaling	2.02	1.57	2.04	2.33
Huntington's Disease Signaling	2.02	1.62	2.04	3.73
IL-8 Signaling	1.99	1.55	1.18	3.74
BAG2 Signaling Pathway	1.84	1.84	2.52	1.8
Bladder Cancer Signaling	1.84	1.83	1.85	2.71
Airway Pathology in Chronic Obstructive Pulmonary Disease	1.8	1.8	1.82	0.807
FGF Signaling	1.8	1.8	1.82	1.76
Triacylglycerol Degradation	1.79	2.55	1.75	1.93
Glutamate Removal from Folates	1.77	1.77	1.77	1.62
Colorectal Cancer Metastasis Signaling	1.74	1.36	1.38	2.6
Phagosome Maturation	1.71	1.71	1.27	2.78
γ-glutamyl Cycle	1.7	1.7	1.71	3.7
Osteoarthritis Pathway	1.69	1.69	2.15	1.22
Human Embryonic Stem Cell Pluripotency	1.62	1.18	1.2	1.32
Notch Signaling	1.58	1.57	1.58	1.2
Adenosine Nucleotides Degradation II	1.53	1.53	1.53	0.491
Chondroitin Sulfate Degradation (Metazoa)	1.53	1.53	2.64	1.26
Wound Healing Signaling Pathway	1.53	1.93	1.55	2.48
White Adipose Tissue Browning Pathway	1.51	1.5	1.52	0.372
Dermatan Sulfate Degradation (Metazoa)	1.48	1.48	2.56	1.21
MSP-RON Signaling in Cancer Cells Pathway	1.48	1.48	1.05	2.16
Asparagine Degradation I	1.47	1.47		1.32
Ephrin B Signaling	1.46	0.903	0.914	4.25
IL-10 Signaling	1.46	1.46	1.47	0.284
Glioma Invasiveness Signaling	1.44	1.44	1.45	3.44
B Cell Development	1.41	0.762	1.42	1.05
Caveolar-mediated Endocytosis Signaling	1.41	1.4	0.441	3.36
Purine Nucleotides Degradation II (Aerobic)	1.39	1.39	1.39	0.432
PTEN Signaling	1.36	1.36	1.85	2.46
Semaphorin Neuronal Repulsive Signaling Pathway	1.35	0.932	1.36	5.41
Protein Kinase A Signaling	1.31	1.31	1.65	2.29
Epithelial Adherens Junction Signaling	1.28	1.28	1.29	7.32
Ovarian Cancer Signaling	1.27	1.27	1.28	2.8
Hepatic Fibrosis Signaling Pathway	1.24	1.23	0.983	3.21
Inhibition of ARE-Mediated mRNA Degradation Pathway	1.24	1.23	1.25	2.24
Glioma Signaling	1.21	0.79	0.801	5.03
Crosstalk between Dendritic Cells and Natural Killer Cells	1.16	1.15	1.72	0.754
Glutathione Redox Reactions I	1.09	0.417	1.09	3.31
Estrogen Receptor Signaling	1.04	0.578	1.05	4.23
GDP-mannose Biosynthesis	1.01	1.01	1.01	2.09
IGF-1 Signaling	0.991	0.582	1	3.04
Phospholipases	0.975	1.56	0.983	3.7
Glutathione-mediated Detoxification	0.883	0.327	0.889	3.63
Phagosome Formation	0.873	0.688	0.893	5.65
Cholecystokinin/Gastrin-mediated Signaling	0.836	0.836	0.487	4.51
Thyroid Cancer Signaling	0.818	0.408	0.827	2.53
RHOA Signaling	0.793	0.45	0.457	8.12

Canonical Pathways (NO)	Original	A1	A2	A3
Glioblastoma Multiforme Signaling	0.775	0.476	0.484	4.76
Pancreatic Adenocarcinoma Signaling	0.775	0.772	0.783	1.95
Sucrose Degradation V (Mammalian)	0.764	0.762	0.767	1.56
HIPPO signaling	0.752	1.24	0.759	2.36
Protein Ubiquitination Pathway	0.721	1	1.02	3.41
Ephrin A Signaling	0.719	0.719	0.726	2.28
Colanic Acid Building Blocks Biosynthesis	0.67	0.67	0.674	1.36
Fcγ Receptor-mediated Phagocytosis in Macrophages and Monocytes	0.666	0.664	0.325	3.36
PI3K Signaling in B Lymphocytes	0.644	0.355	0.652	3.74
Endothelin-1 Signaling	0.638	0.382	0.389	3.62
Actin Cytoskeleton Signaling	0.62	0.616	0.399	11.8
EGF Signaling	0.618	0.618	0.623	1.37
CSDE1 Signaling Pathway	0.607	0.208	0.613	1.34
RAN Signaling	0.597	0.595	0.599	2.14
PPAR Signaling	0.561	0.955	0.266	1.35
Telomerase Signaling	0.561	0.559	0.567	2.36
Semaphorin Signaling in Neurons	0.545	0.544	0.55	2.43
Autophagy	0.517	0.3	1.13	1.45
Role of Tissue Factor in Cancer	0.5	0.498	0.506	2.71
Neuregulin Signaling	0.493	0.492	0.499	2.13
Remodeling of Epithelial Adherens Junctions	0.491	0.489		2.23
SPINK1 General Cancer Pathway	0.483	0.481	0.488	1.6
Agrin Interactions at Neuromuscular Junction	0.475	1.5	0.939	3.57
Sphingosine-1-phosphate Signaling	0.475	0.474		4.48
Tumoricidal Function of Hepatic Natural Killer Cells	0.471	1.2	0.474	4.76
ERK5 Signaling	0.444	0.442	0.449	2.06
IL-6 Signaling	0.429	0.427	0.434	2.42
Leptin Signaling in Obesity	0.429	0.428	0.434	3.32
Angiopoietin Signaling	0.423	0.421	0.427	4.02
NRF2-mediated Oxidative Stress Response	0.423		0.429	1.91
Chemokine Signaling	0.402	0.401		7.25
Renal Cell Carcinoma Signaling	0.402	0.401	0.406	3.17
Estrogen-Dependent Breast Cancer Signaling	0.396		0.4	1.87
RAC Signaling	0.379	0.377		3.28
VEGF Family Ligand-Receptor Interactions	0.377	0.376	0.381	2.39
Reelin Signaling in Neurons	0.375	0.373	0.379	1.71
D-myo-inositol-5-phosphate Metabolism	0.372	0.37	0.613	1.7
Insulin Receptor Signaling	0.37	0.368	0.674	1.7
Apelin Endothelial Signaling Pathway	0.365			2.14
PDGF Signaling	0.365	0.364		3.65
BMP signaling pathway	0.36	0.358	0.363	1.73
Xenobiotic Metabolism AHR Signaling Pathway	0.36	0.358	0.363	1.73
Inhibition of Angiogenesis by TSP1	0.355	0.354	0.357	1.32
Regulation of Cellular Mechanics by Calpain Protease	0.348	0.347	0.352	2.25
Acute Myeloid Leukemia Signaling	0.337	0.335		1.65
ERBB Signaling	0.321	0.321	0.325	4.05
Non-Small Cell Lung Cancer Signaling	0.321	0.321	0.325	2.71
Factors Promoting Cardiogenesis in Vertebrates	0.315	0.313	0.32	1.92

Canonical Pathways (NO)	Original	A1	A2	A3
Oncostatin M Signaling	0.282	0.281	0.284	2.45
G Protein Signaling Mediated by Tubby	0.275			1.68
Coronavirus Replication Pathway	0.268	0.268	0.754	4.98
CXCR4 Signaling	0.259			4.82
Paxillin Signaling	0.259	0.258		2.92
Gαq Signaling	0.256			2.08
Antioxidant Action of Vitamin C	0.243	0.243	0.246	2.24
CDK5 Signaling	0.236	0.511	0.519	2.2
UVC-Induced MAPK Signaling	0.234	0.233	0.236	1.47
CNTF Signaling	0.204	0.203	0.206	2.62
14-3-3-mediated Signaling				3
Acetyl-CoA Biosynthesis III (from Citrate)				1.62
Actin Nucleation by ARP-WASP Complex				5.63
Adrenomedullin signaling pathway				2.48
Apelin Cardiomyocyte Signaling Pathway				3.19
Apoptosis Signaling				2.44
Calcium Signaling				1.77
Cardiac Hypertrophy Signaling				4.71
CCR3 Signaling in Eosinophils				4.63
Cellular Effects of Sildenafil (Viagra)				4.18
Chronic Myeloid Leukemia Signaling				2.36
Death Receptor Signaling				2.66
D-myo-inositol (1,4,5)-Trisphosphate Biosynthesis				1.62
EIF2 Signaling				1.71
Endometrial Cancer Signaling				1.84
ERB2-ERBB3 Signaling				1.7
ERBB4 Signaling				2.23
ERK/MAPK Signaling				1.8
Fc Epsilon RI Signaling				1.62
FcγRIIB Signaling in B Lymphocytes			0.375	3
Ferroptosis Signaling Pathway				1.39
FLT3 Signaling in Hematopoietic Progenitor Cells				1.32
fMLP Signaling in Neutrophils				2.89
G Beta Gamma Signaling				2.39
Gap Junction Signaling				2.06
GDNF Family Ligand-Receptor Interactions			0.86	5.71
Gluconeogenesis I				2.49
Glycolysis I				2.43
GM-CSF Signaling				1.58
GNRH Signaling				2.16
GP6 Signaling Pathway		0.433	0.775	3
Gα12/13 Signaling				4.7
HER-2 Signaling in Breast Cancer			0.271	2.04
HGF Signaling				2.33
Hypoxia Signaling in the Cardiovascular System				1.44
ICOS-ICOSL Signaling in T Helper Cells				1.63
IL-2 Signaling				1.81
IL-3 Signaling				1.92
ILK Signaling				2.46

Canonical Pathways (NO)	Original	A1	A2	A3
Induction of Apoptosis by HIV1				1.7
Integrin Signaling				6.61
JAK/STAT Signaling				1.32
LPS-stimulated MAPK Signaling				1.78
Melanoma Signaling				1.5
Melatonin Signaling				1.53
Molecular Mechanisms of Cancer				2.21
mTOR Signaling				2.73
MYC Mediated Apoptosis Signaling				1.5
Neurotrophin/TRK Signaling				2.57
Neurovascular Coupling Signaling Pathway				1.33
NF-κB Activation by Viruses				1.4
NGF Signaling				2.6
Oxytocin In Brain Signaling Pathway				1.31
Oxytocin Signaling Pathway				6.43
P2Y Purigenic Receptor Signaling Pathway				1.82
p70S6K Signaling				4.73
PAK Signaling				6.01
PEDF Signaling				3.03
Phospholipase C Signaling				1.85
PPARα/RXRα Activation		0.37		2.11
Prolactin Signaling				1.76
Prostate Cancer Signaling				2.2
Regulation of Actin-based Motility by Rho				4.63
Regulation of eIF4 and p70S6K Signaling				2.4
Renin-Angiotensin Signaling				2.03
RHOGDI Signaling				3.63
Role of JAK1 and JAK3 in γc Cytokine Signaling				1.6
Role of MAPK Signaling in the Pathogenesis of Influenza				2.44
Role of NFAT in Cardiac Hypertrophy				2.11
Signaling by Rho Family GTPases				3.08
SNARE Signaling Pathway				2.24
Sumoylation Pathway				1.42
Synaptic Long-Term Depression				2.55
Synaptic Long-Term Potentiation				2.35
Thrombin Signaling				6.23
Thrombopoietin Signaling				1.75
Tight Junction Signaling				2.4
TNFR1 Signaling				3.63
UVA-Induced MAPK Signaling				2.6
VEGF Signaling				2.57
Virus Entry via Endocytic Pathways				2.44
WNT/Ca+ pathway				1.67
Xenobiotic Metabolism General Signaling Pathway				2.11
α-Adrenergic Signaling				1.33

Footnote: Shown values = -log(p) for each predicted canonical pathway. Only significant predictions with -log(p) > 1.3 (i.e., $p < 0.05$) were shown; however, when significance was reached in at least one of the datasets (original or amplified), non-significant predictions with -log(p) values < 1.3 were shown for comparison. Blank means no prediction.

Table S3A: Diseases & Functions analysis for enriched immuno-inflammatory processes in integrated multi-omics datasets for the T1D high-risk (HR) subject group.

Diseases & Functions (HR)	Original	A1	A2	A3
Engulfment of cells	+++	+++	+++	+++
Phagocytosis	+++	+++	+++	+++
Phagocytosis of cells	+++	+++	+++	+++
Migration of phagocytes	+++	+++		+++
Synthesis of reactive oxygen species	+++	+++	+	+++
Cell movement of leukocytes	+++	+++	+++	+++
Cell movement of phagocytes	+++	+++	++	+++
Leukocyte migration	+++	+++		+++
Production of reactive oxygen species	+++	+++		
Transmigration of cells	+++	+++	++	+++
Cell movement of dendritic cells	+++	+++		
Migration of antigen presenting cells	+++	+++		
Cell movement of antigen presenting cells	+++	+++	++	+++
Internalization of cells	+++	+++		+++
Adhesion of immune cells	+++	+++	+++	++
Generation of reactive oxygen species	+++	+++	+++	+++
Engulfment of leukocytes	+++	+++	+++	+++
Migration of myeloid cells	+++	+++	+++	+++
Activation of cells	+++	+++		+++
Cell movement of myeloid cells	+++	+++	++	+++
Activation of leukocytes	+++	+++	+++	+++
Immune response of myeloid cells	+++	+++		+++
Activation of mononuclear leukocytes	+++	+++	+++	
Migration of dendritic cells	+++	+++		
Cell movement of mononuclear leukocytes	+++	+++	+++	+++
Transmigration of leukocytes	+++	+++		+++
Production of hydrogen peroxide	+++	+++	++	+++
Engulfment of antigen presenting cells	+++	+++	++	+++
Engulfment of phagocytes	+++	+++	+++	+++
Phagocytosis of leukocytes	+++	+++	- -	+++
Production of superoxide	+++	+++	+	+++
Phagocytosis of myeloid cells	+++	+++		+++
Immune response of phagocytes	+++	+++	+	+++
Proliferation of CD4+ T-lymphocytes	+++	+++	+++	+++
Biosynthesis of hydrogen peroxide	+++	+++	+++	+++
Cell movement of lymphocytes	+++	+++		+++
Generation of superoxide	+++	+++	+++	+++
Immune response of cells	+++	+++	+++	+++
Activation of lymphatic system cells	+++	+++		

Diseases & Functions (HR)	Original	A1	A2	A3
Concentration of eicosanoid	+++	+++	+++	+++
Phagocytosis of antigen presenting cells	+++	+++	++	+++
Immune response of macrophages	+++	+++		+++
Phagocytosis of phagocytes	+++	+++	+++	+++
Chemotaxis	+++	+++		+++
Vascularization	+++	+++		+++
Immune response of leukocytes	+++	+++	+	+++
Inflammatory response	+++	+++	+++	+++
Angiogenesis	+++	+++		+++
Development of vasculature	+++	+++		+++
Quantity of reactive oxygen species	+++	+++	+++	+++
Activation of T lymphocytes	+++	+++	++	+++
Proliferation of immune cells	+++	++	++	+++
Metabolism of hydrogen peroxide	+++	+++		+++
Phagocytosis by macrophages	+++	+++	+++	+++
Neovascularization	+++	+++	++	+++
Binding of antigen presenting cells	++		+++	
Migration of cells	++	+++		+++
Cell movement of granulocytes	++	++	+++	++
Migration of mononuclear leukocytes	++	++		+++
Concentration of prostaglandin	++	++		
Activation of myeloid cells	++	++	++	+++
Cell movement	++	++		+++
Neovascularization of organ	++	++		+++
Homing of leukocytes	++	++		+++
Activation of lymphocytes	++	+++	++	++
Vascularization of eye	++	++		
Activation of phagocytes	++	+++	++	+++
Binding of phagocytes	++	++		
Cell proliferation of T lymphocytes	++	++		+++
Cell death of phagocytes	++	++	++	++
Chemotaxis of phagocytes	++	++		+++
Proliferation of lymphocytes	++	++	+++	+++
Binding of myeloid cells	++	++		+++
Chemotaxis of leukocytes	++	++		+++
Neovascularization of eye	++	++		
Metabolism of reactive oxygen species	++	++		+++
Binding of professional phagocytic cells	++	++		
Transmigration of mononuclear leukocytes	++	++	++	
Lymphocyte migration	++	++		++
Activation of macrophages	++	++	+	++
Cell death of antigen presenting cells	++	++	+	++

Diseases & Functions (HR)	Original	A1	A2	A3
Cellular infiltration	++	++		+
Homing of lymphatic system cells	++	++		
Adhesion of phagocytes	++			
Release of reactive oxygen species	++	++	++	++
Adhesion of leukocyte cell lines	++	++		
Formation of reactive oxygen species	++	++	++	++
Proliferation of mononuclear leukocytes	++	++		+++
Cellular infiltration by leukocytes	++	++	-	
Cell movement of macrophages	++	++	+	++
Chemotaxis of myeloid cells	++	++		+++
Degranulation of cells	++			+
Proliferation of lymphatic system cells	++	++	+	+++
Cell movement of T lymphocytes	++	++	++	
Oxidative stress response of cells	++	++		
Cell movement of monocytes	++	++	++	
Quantity of leukocytes	+	+	++	+++
Cellular infiltration by macrophages	+	+		
Quantity of antigen presenting cells	+	++		
Synthesis of nitric oxide	+	+	+	+
Quantity of lymphocytes	+	+	+++	+++
Cell death of immune cells	+		+	+
Accumulation of granulocytes	+	+	+++	
Ferroptosis	+	+		+
Quantity of nitric oxide	+	+		
Inhibition of leukocytes	+	+	-	
Glucose metabolism disorder	+	-	+	+
Quantity of lymphatic system cells	+	+	++	+++
Accumulation of leukocytes	+	+	+	
Accumulation of myeloid cells	-	-		
Accumulation of phagocytes	-	-		
Diabetes mellitus	-	-		-
Inflammation of body cavity	-	- - -	+	-
Systemic autoimmune syndrome	- -	-	-	- -
Inflammation of absolute anatomical region	- -	- -	+	-
Quantity of myeloid cells	- -	- -		- -
Inflammation of organ	- -	- -	--	- -
Immune mediated inflammatory disease	- -	- -		-
Cell death of B-lymphocyte derived cell lines	- -	- -		- -
Depletion of T lymphocytes	- - -	- - -		
Hematopoiesis of mononuclear leukocytes				+++
Quantity of mononuclear leukocytes			++	+++
Quantity of T lymphocytes				+++

Diseases & Functions (HR)	Original	A1	A2	A3
Cell movement of lymphatic system cells				+++
Recruitment of myeloid cells				+++
Recruitment of cells			++	+++
Engulfment of myeloid cells			+++	+++
Response of antigen presenting cells			++	+++
Response of phagocytes				+++
Interaction of leukocytes				+++
Binding of leukocytes				+++
Response of macrophages			+++	+++
Immune response of antigen presenting cells		+++	+++	+++
Response of myeloid cells				+++
Chemotaxis of mononuclear leukocytes				+++
Synthesis of eicosanoid		+++		+++
Shape change of leukocytes				+++
Homing of mononuclear leukocytes				+++
Cell death of macrophages		+++	+++	+++
Metabolism of prostaglandin				+++
Synthesis of prostaglandin				+++
Apoptosis of beta islet cells		++	++	++
Cell movement of neutrophils			++	++
Function of leukocytes				++
Degranulation of phagocytes				+
Degranulation of leukocytes				+
Degranulation of myeloid cells				-
Differentiation of myeloid leukocytes			- -	-
Inflammation of joint		- - -	- - -	-
Autophagy of cells			+	-
Quantity of phagocytes		-		
Release of nitric oxide		+		
Cellular infiltration by phagocytes		+		
Response of mononuclear leukocytes		++		
Cell death of myeloid cells		+++		
Function of antigen presenting cells			- - -	
Release of hydrogen peroxide			-	
Function of macrophages			-	
Transmigration of myeloid cells			++	
Transmigration of phagocytes			++	
Activation of myeloid-derived suppressor cells			+++	
Activation-induced cell death of CD4+ T-lymphocytes			+++	

Footnote: z-score values: +++ ≥2; ++ ≥1, <2; + ≥0, <1; - >-1, <0; - - ≤-1, >-2; - - - ≤-2; blank means no prediction.

Table S3B: Diseases & Functions analysis for enriched immuno-inflammatory processes in integrated multi-omics datasets for the T1D new-onset (NO) subject group.

Diseases & Functions (NO)	Original	A1	A2	A3
Cell movement	+++	+++	+++	+++
Migration of cells	+++	+++	+++	+++
Engulfment of cells	+++	+++	+++	+++
Phagocytosis	+++	+++	+++	+++
Phagocytosis of cells	+++	+++	+++	+++
Development of vasculature	+++	+++	+++	+++
Angiogenesis	+++	+++	+++	++
Internalization of cells	+++	+++	+++	+++
Leukocyte migration	+++	+++	+++	+++
Cell movement of leukocytes	+++	+++	+++	+++
Chemotaxis	+++	+++	+++	+++
Cell movement of phagocytes	+++	+++	+++	+++
Migration of phagocytes	+++	+++	+++	
Transmigration of cells	+++	+++	+++	+++
Engulfment of leukocytes	+++	+++	+++	+++
Engulfment of phagocytes	+++	+++	+++	+++
Immune response of cells	+++	+++	+++	+++
Binding of leukocytes	+++	+++	+++	+++
Activation of mononuclear leukocytes	+++	+++	+++	
Engulfment of myeloid cells	+++	+++	+++	+++
Activation of cells	+++	+++		
Cell movement of mononuclear leukocytes	+++	+++	+++	+++
Engulfment of antigen presenting cells	+++	+++	+++	+++
Cell movement of myeloid cells	+++	+++	+++	+++
Migration of myeloid cells	+++	+++	+++	+++
Synthesis of reactive oxygen species	+++	+++	+++	+++
Phagocytosis of phagocytes	+++	+++	+++	+++
Transmigration of leukocytes	+++	+++	+++	
Adhesion of immune cells	+++	+++	+++	+++
Phagocytosis of myeloid cells	+++	+++	+++	+++
Production of reactive oxygen species	+++	+++		
Phagocytosis of antigen presenting cells	+++	+++	+++	+++
Activation of leukocytes	+++	+++	+++	+++
Activation of lymphatic system cells	+++	+++	+++	
Phagocytosis of blood cells	+++	+++	+++	+++
Response of phagocytes	+++	+++	+++	+++
Cell movement of antigen presenting cells	+++	+++	+++	+++
Immune response of myeloid cells	+++	+++	+++	+++
Immune response of phagocytes	+++	+++	+++	+++

Diseases & Functions (NO)	Original	A1	A2	A3
Proliferation of mononuclear leukocytes	+++	+++	+++	+++
Proliferation of immune cells	+++	+++	+++	+++
Response of myeloid cells	+++	+++	+++	+++
Phagocytosis by macrophages	+++	+++	+++	+++
Proliferation of lymphatic system cells	+++	+++	+++	+++
Immune response of leukocytes	+++	+++	+++	+++
Quantity of lymphocytes	+++	+++	+++	+++
Response of antigen presenting cells	+++	+++	+++	+++
Inflammatory response	+++	+++	+++	+++
Proliferation of lymphocytes	+++	+++	+++	+++
Cell movement of monocytes	+++	+++	+++	
Generation of reactive oxygen species	+++	+++	+++	+++
Cell proliferation of T lymphocytes	+++	+++	+++	++
Migration of mononuclear leukocytes	+++	+++	+++	+++
Quantity of lymphatic system cells	+++	+++	+++	+++
Production of superoxide	+++	+++	+++	+++
Vascularization	+++	+++	+++	
Proliferation of CD4+ T-lymphocytes	+++	+++	+++	+++
Immune response of antigen presenting cells	+++		+++	+++
Quantity of mononuclear leukocytes	+++	+++	+++	
Production of hydrogen peroxide	+++	+++	+++	+++
Activation of myeloid cells	+++	+++	+++	+++
Synthesis of eicosanoid	+++	+++		
Concentration of eicosanoid	+++	+++	+++	+++
Cell movement of granulocytes	+++	+++	+++	+++
Response of macrophages	+++	+++	+++	+++
Immune response of macrophages	+++	+++	+++	
Biosynthesis of hydrogen peroxide	+++	+++	+++	+++
Recruitment of cells	+++	+++	+++	+++
Activation of lymphocytes	+++	+++	+++	++
Generation of superoxide	+++	+++	+++	+++
Binding of myeloid cells	+++	+++	+++	+++
Metabolism of eicosanoid	+++			
Metabolism of reactive oxygen species	+++	+++	+++	+++
Cell movement of lymphatic system cells	+++	++		+++
Quantity of reactive oxygen species	++	+++	+++	+++
Cell death of macrophages	++	+++	+++	+++
Concentration of prostaglandin	++	++		
Stimulation of leukocytes	++			
Cell movement of lymphocytes	++	++		+++
Metabolism of hydrogen peroxide	++	+++	+++	+++
Neovascularization	++	++	+++	

Diseases & Functions (NO)	Original	A1	A2	A3
Activation of phagocytes	++	+++	+++	+++
Activation of T lymphocytes	++	+++	+++	+++
Cell death of phagocytes	++	++	++	++
Binding of phagocytes	++	++	+++	
Neovascularization of organ	++	++		
Binding of professional phagocytic cells	++	++	+++	+++
Activation of macrophages	++	++	++	++
Cell death of antigen presenting cells	++	++	++	++
Cell movement of macrophages	++	++	++	
Quantity of leukocytes	++	++	++	+++
Degranulation of myeloid cells	++	++		+
Degranulation of leukocytes	++	++		+
Formation of reactive oxygen species	++	++	++	++
Vascularization of eye	++	+		
Neovascularization of eye	++	+		
Cellular infiltration by leukocytes	++	++	+	++
Cellular infiltration by leukocytes	++	++	+	++
Oxidative stress response of cells	++	++		
Accumulation of leukocytes	++	++	+	
Release of reactive oxygen species	++	++	++	++
Degranulation of cells	+	+		+
Cellular infiltration	+	+	+	++
Function of leukocytes	+	+	+	+
Synthesis of nitric oxide	+	+	+	+
Degranulation of phagocytes	+	+		+
Release of nitric oxide	+	+		
Ferroptosis	+	+	+	+
Quantity of nitric oxide	+	+		
Inhibition of leukocytes	+	+	+	
Cell death of immune cells	-		+	+
Diabetes mellitus	-	-	-	-
Systemic autoimmune syndrome	-	+	-	-
Accumulation of phagocytes	-	-	-	
Accumulation of myeloid cells	-	-	-	
Complement activation	-	-		+
Inflammation of absolute anatomical region	-	-	-	-
Quantity of myeloid cells	-	- -	-	
Inflammation of body cavity	- -	- -	-	-
Inflammation of organ	- -	- -	- -	- -
Immune mediated inflammatory disease	- -	- -	- -	-
Cell death of B-lymphocyte derived cell lines	- -	- -	- -	- -
Apoptosis of leukocytes	- - -	- -	- -	

Diseases & Functions (NO)	Original	A1	A2	A3
Migration of lymphatic system cells				+++
Lymphocyte migration				+++
Transmigration of phagocytes		+++	+++	+++
Cell movement of neutrophils		++	++	+++
Interaction of leukocytes				+++
Recruitment of leukocytes				+++
Binding of antigen presenting cells			+++	+++
Recruitment of phagocytes				+++
Transmigration of mononuclear leukocytes		+++	+++	+++
Transmigration of mononuclear leukocytes		+++	+++	+++
Homing of leukocytes				+++
Interaction of phagocytes				+++
Chemotaxis of leukocytes				+++
Secretion of molecule				+++
Homing of mononuclear leukocytes				+++
Chemotaxis of phagocytes				+++
Chemotaxis of antigen presenting cells				+++
Activation of antigen presenting cells				+++
Chemotaxis of myeloid cells				+++
Apoptosis of beta islet cells		++	++	++
Differentiation of myeloid leukocytes				+
Inflammation of joint		- -	- - -	+
Rheumatic Disease		- - -	- - -	-
Inflammation of central nervous system		- -	- -	- -
Accumulation of granulocytes		-		
Transmigration of myeloid cells			++	
Cellular infiltration by macrophages		+		
Cellular infiltration by phagocytes		+		
Cell death of myeloid cells		+++		
Autophagy of cells			-	

Footnote: z-scores: +++ ≥2; ++ ≥1, <2; + ≥0, <1; - >-1, <0; - - ≤-1, >-2; - - - ≤-2; blank means no prediction.

Table S4A: Lists of consolidated candidate biomarkers predicted by *IPA* in the context of the shown Diseases&Functions in the proteomics and metabolomics datasets for the T1D high-risk (HR) subject group.

Diseases & Functions (HR)	Original		A1		A2		A3	
	p	Biomarker name*	*p*	Biomarker name*	*p*	Biomarker name*	*p*	Biomarker name*
**Diabetes mellitus**	3.53E-13	APOA2, APOE, CD44, CETP, GPNMB, IGF1, IGFBP2, JAG1, L1CAM, LDLR, MEP1B, MMP14, MMP2, MMP9, PTGDS, PTPRC, SELL, SFTPD, VCAM1	4.69E-14	APOA2, APOE, CD44, CETP, GPNMB, IGF1, IGFBP2, IGHM, JAG1, L1CAM, LDLR, MEP1B, MMP14, MMP2, MMP9, PTGDS, PTPRC, SELL, SFTPD, VCAM1	6.1E-15	ANXA1, APOA2, APOE, CD44, CETP, FGFR1, GPNMB, IGF1, IGFBP2, IGHM, JAG1, L1CAM, LDLR, MEP1B, MMP14, MMP2, MMP9, PTGDS, SELL, SFTPD, VCAM1	1.91E-25	ACE, ADK, AKT1, APOA1, APOA4, APOB, APOC1, CASP3, CCL5, CD36, CETP, CR2, CXCL12, EGFR, FADD, FAS, FGFR1, GFAP, GSTO1, GSTP1, HPSE, HSPB1, IGF1, IGF2, IGHM, IL18, L1CAM, MASP2, MMP14, MMP9, MSTN, PCSK9, PDE5A, PON1, PTGDS, PTPRC, RETN, SFTPD, SOD1, SRC, VCAM1
**Chemotaxis**	5.25E-12	APOE, CD44, CSF1R, IGF1, L1CAM, LDLR, MMP14, MMP2, MMP9, PTPRC, SELL, SFTPD, VCAM1	8.13E-12	APOE, CD44, CSF1R, IGF1, L1CAM, LDLR, MMP14, MMP2, MMP9, PTPRC, SELL, SFTPD, VCAM1	1.24E-11	ANXA1, APOE, CD44, CSF1R, IGF1, L1CAM, LDLR, MMP14, MMP2, MMP9, SELL, SFTPD, VCAM1	3.95E-30	AKT1, APOA1, CCL5, CD36, CD9, CSF1, CXCL12, EFNB2, EGF, EGFR, FADD, FAS, HDAC6, HPSE, HSPB1, IGF1, IGF2, IL18, KIT, L1CAM, MET, MMP14, MMP9, MSTN, NRP1, NTRK3, PIGR, PTPRC, RTN4, SAA1, SFTPD, SRC, VCAM1
**Cell movement**	1.7E-18	APOE, CD44, CSF1R, CUL1, FGFR2, FSCN1, GPNMB, IGF1, IGFBP2, JAG1, L1CAM, LDLR, LYVE1, MEP1B, MMP14, MMP2, MMP9, NCAM1, PRDX1, PRDX6, PROS1, PTGDS, PTPRC, PTPRG, SELL, SFTPD, TIMP2, VCAM1	2.96E-19	APOE, CD44, CSF1R, CUL1, FGFR2, FSCN1, GPNMB, IGF1, IGFBP2, IGHM, JAG1, L1CAM, LDLR, LYVE1, MEP1B, MMP14, MMP2, MMP9, NCAM1, OSMR, PRDX1, PRDX6, PTGDS, PTPRC, PTPRG, SELL, SFTPD, TIMP2, VCAM1	5.13E-20	AIMP1, ANXA1, APOE, CD44, CSF1R, CUL1, FGFR1, FGFR2, FSCN1, GPNMB, IGF1, IGFBP2, IGHM, JAG1, L1CAM, LDLR, LYVE1, MEP1B, MMP14, MMP2, MMP9, NCAM1, PRDX1, PRDX6, PTGDS, PTPRG, SELL, SFTPD, TIMP2, VCAM1	1.9E-40	AKT1, ALDOA, APOA1, APOB, APOC1, CCL5, CD14, CD36, CD9, COMP, CR2, CSF1, CXCL12, EFNB2, EGF, EGFR, FADD, FAS, FGFR1, FLNC, FSCN1, GFAP, HDAC6, HPSE, HSPB1, IGF1, IGF2, IGHM, IL18, KIT, KLK6, L1CAM, LCAT, LDHC, LYVE1, MASP2, MET, MMP14, MMP9, MSTN, NRP1, NTRK3, OSMR, PCSK9, PIGR, PRDX1, PROC, PTGDS, PTPRC, PTPRG, RET, RETN, RTN4, SAA1, SFTPD, SOD1, SRC, STAB2, TLN1, TPM3, USP9X, VASP, VCAM1, ZYX
**Migration of cells**	6.09E-17	APOE, CD44, CSF1R, CUL1, FGFR2, FSCN1, GPNMB, IGF1, IGFBP2, JAG1, L1CAM, LDLR, LYVE1, MEP1B, MMP14, MMP2, MMP9, NCAM1, PRDX1, PROS1, PTGDS, PTPRC, SELL, SFTPD, TIMP2, VCAM1	2.07E-16	APOE, CD44, CSF1R, CUL1, FGFR2, FSCN1, GPNMB, IGF1, IGFBP2, JAG1, L1CAM, LDLR, LYVE1, MEP1B, MMP14, MMP2, MMP9, NCAM1, OSMR, PRDX1, PTGDS, PTPRC, SELL, SFTPD, TIMP2, VCAM1	3.59E-17	AIMP1, ANXA1, APOE, CD44, CSF1R, CUL1, FGFR1, FGFR2, FSCN1, GPNMB, IGF1, IGFBP2, JAG1, L1CAM, LDLR, LYVE1, MEP1B, MMP14, MMP2, MMP9, NCAM1, PRDX1, PTGDS, SELL, SFTPD, TIMP2, VCAM1	1.23E-37	AKT1, ALDOA, APOA1, APOB, APOC1, CCL5, CD14, CD36, CD9, COMP, CR2, CSF1, CXCL12, EFNB2, EGF, EGFR, FAS, FGFR1, FLNC, FSCN1, GFAP, HDAC6, HPSE, HSPB1, IGF1, IGF2, IL18, KIT, KLK6, L1CAM, LCAT, LYVE1, MASP2, MET, MMP14, MMP9, MSTN, NRP1, NTRK3, OSMR, PCSK9, PIGR, PRDX1, PROC, PTGDS, PTPRC, RET, RETN, RTN4, SAA1, SFTPD, SOD1, SRC, STAB2, TLN1, TPM3, USP9X, VASP, VCAM1, ZYX
**Cellular infiltratio n**	6.91E-10	APOE, CD44, CSF1R, LDLR, MMP14, MMP2, MMP9, PRDX1, PRDX6, PTGDS, SELL, VCAM1	6.3E-11	APOE, CD44, CSF1R, IGHM, LDLR, MMP14, MMP2, MMP9, PRDX1, PRDX6, PTGDS, SELL, VCAM1	5.49E-12	ANXA1, APOE, CD44, CSF1R, IGHM, LDLR, MMP14, MMP2, MMP9, PRDX1, PRDX6, PTGDS, SELL, VCAM1	1.75E-21	AKT1, ALDOA, APOA1, CCL5, CD14, CD36, CSF1, CXCL12, EGFR, FAS, GFAP, HSPB1, IGHM, IL18, LCAT, MASP2, MET, MMP14, MMP9, NRP1, PCSK9, PRDX1, PROC, PTGDS, RETN, SAA1, VASP, VCAM1
**Accumulat ion of cells**	5.02E-10	APOE, CD44, FGFR2, IGF1, LDLR, MMP9, PTGDS, SELL, TIMP2, VCAM1	2.99E-11	APOE, CD44, FGFR2, IGF1, LDLR, MMP9, OSMR, PTGDS, SELL, TIMP2, VCAM1	5.87E-14	AIMP1, ANXA1, APOE, CD44, FGFR1, FGFR2, IGF1, LDLR, MMP9, PTGDS, SELL, TIMP2, VCAM1	2.07E-17	APOA1, CCL5, CSF1, CXCL12, EGF, EGFR, FADD, FAS, FGFR1, IGF1, IL18, KIT, MBL2, MMP9, OSMR, PTGDS, RET, SOD1, TLN1, VCAM1

Diseases & Functions (HR)	Original		A1		A2		A3	
	p	Biomarker name*	*p*	Biomarker name*	*p*	Biomarker name*	*p*	Biomarker name*
Quantity of lymphocytes	6.62E-13	APOE, CD44, CSF1R, FGFR2, IGF1, IGKC, JAG1, LDLR, MMP9, MSH2, PRDX1, PTPRC, SELL, SFTPD, VCAM1	5.59E-14	APOE, CD44, CSF1R, FGFR2, IGF1, IGHM, IGKC, JAG1, LDLR, MMP9, MSH2, PRDX1, PTPRC, SELL, SFTPD, VCAM1	4.58E-15	ANXA1, APOE, CD44, CSF1R, FGFR1, FGFR2, IGF1, IGHM, IGKC, JAG1, LDLR, MMP9, MSH2, PRDX1, SELL, SFTPD, VCAM1	3.85E-21	AKT1, APOA1, CASP3, CCL5, CD36, CR2, CSF1, CXCL12, EFNB2, FADD, FAS, FGFR1, GSTP1, IGF1, IGF2, IGHM, IL18, KIT, MBL2, MMP9, PIGR, PRDX1, PROC, PTPRC, SAA1, SFTPD, SOD1, TLN1, VCAM1
Quantity of myeloid cells	4.11E-09	APOE, CSF1R, IGF1, LDLR, MMP9, PRDX1, PROS1, SELL, SFTPD, TIMP2, VCAM1	N/A	N/A	8.14E-09	ANXA1, APOE, CSF1R, IGF1, LDLR, MMP9, PRDX1, SELL, SFTPD, TIMP2, VCAM1	1.45E-16	APOB, CD36, CD9, CR2, CSF1, CXCL12, FADD, FAS, HPSE, IGF1, IL18, KIT, LCAT, MBL2, MMP9, PRDX1, PROC, SAA1, SFTPD, SOD1, SRC, VASP, VCAM1
Quantity of T lymphocytes	8.48E-11	APOE, CD44, FGFR2, IGF1, JAG1, LDLR, MMP9, PRDX1, PTPRC, SELL, SFTPD, VCAM1	6.43E-12	APOE, CD44, FGFR2, IGF1, IGHM, JAG1, LDLR, MMP9, PRDX1, PTPRC, SELL, SFTPD, VCAM1	4.65E-13	ANXA1, APOE, CD44, FGFR1, FGFR2, IGF1, IGHM, JAG1, LDLR, MMP9, PRDX1, SELL, SFTPD, VCAM1	2.66E-16	APOA1, CASP3, CCL5, CSF1, EFNB2, FADD, FAS, FGFR1, IGF1, IGF2, IGHM, IL18, KIT, MMP9, PIGR, PRDX1, PROC, PTPRC, SAA1, SFTPD, TLN1, VCAM1
Proliferation of immune cells	3.17E-13	APOE, CD44, CSF1R, CUL1, GPNMB, IGF1, IGFBP2, IGKC, JAG1, LDLR, MMP9, MSH2, PTPRC, SELL, SFTPD, VCAM1	2.94E-14	APOE, CD44, CSF1R, CUL1, GPNMB, IGF1, IGFBP2, IGHM, IGKC, JAG1, LDLR, MMP9, MSH2, PTPRC, SELL, SFTPD, VCAM1	5.33E-14	ANXA1, APOE, CD44, CSF1R, CUL1, GPNMB, IGF1, IGFBP2, IGHM, IGKC, JAG1, LDLR, MMP9, MSH2, SELL, SFTPD, VCAM1	2.87E-18	ADK, AKT1, CASP3, CCL5, CD14, CD36, CR2, CSF1, CXCL12, EFNB2, FADD, FAS, GSTP1, IGF1, IGF2, IGHM, IL18, KIT, MBL2, MET, MMP9, PDE5A, PROC, PTPRC, SFTPD, SRC, TLN1, VCAM1
Proliferation of lymphocytes	2.35E-11	APOE, CD44, CUL1, GPNMB, IGF1, IGFBP2, IGKC, JAG1, MMP9, MSH2, PTPRC, SELL, SFTPD, VCAM1	2.23E-12	APOE, CD44, CUL1, GPNMB, IGF1, IGFBP2, IGHM, IGKC, JAG1, MMP9, MSH2, PTPRC, SELL, SFTPD, VCAM1	3.67E-12	ANXA1, APOE, CD44, CUL1, GPNMB, IGF1, IGFBP2, IGHM, IGKC, JAG1, MMP9, MSH2, SELL, SFTPD, VCAM1	4.16E-15	ADK, AKT1, CASP3, CCL5, CD14, CD36, CR2, CXCL12, EFNB2, FADD, FAS, IGF1, IGF2, IGHM, IL18, KIT, MMP9, PDE5A, PROC, PTPRC, SFTPD, SRC, TLN1, VCAM1
Immune response of cells	1.19E-09	APOA2, APOE, CD44, CSF1R, GSTT1, LDLR, MEP1B, MSH2, PROS1, PTPRC, SFTPD, VCAM1	1.75E-09	APOA2, APOE, CD44, CSF1R, GSTT1, IGHM, LDLR, MEP1B, MSH2, PTPRC, SFTPD, VCAM1	2.53E-09	ANXA1, APOA2, APOE, CD44, CSF1R, GSTT1, IGHM, LDLR, MEP1B, MSH2, SFTPD, VCAM1	2.26E-16	ACE, APOA1, CD14, CD36, CSF1, FADD, FAS, HSPB1, IGHM, IL18, MBL2, MET, PON1, PROC, PTPRC, RET, SAA1, SFTPD, SOD1, SRC, STAB2, TLN1, VASP, VCAM1
Inflammatory response	7.77E-14	APOE, CD44, CSF1R, CUL1, GPX1, IGF1, LDLR, MEP1B, MMP14, MMP2, MMP9, MSH2, PRDX6, PROS1, SELL, SFTPD, VCAM1	1.44E-13	APOE, CD44, CSF1R, CUL1, GPX1, IGF1, IGHM, LDLR, MEP1B, MMP14, MMP2, MMP9, MSH2, PRDX6, SELL, SFTPD, VCAM1	3.12E-17	AIMP1, ANXA1, APOE, CD44, CSF1R, CUL1, FGFR1, GPX1, IGF1, IGHM, LDLR, MEP1B, MMP14, MMP2, MMP9, MSH2, PRDX6, SELL, SFTPD, VCAM1	2.18E-20	ACE, AKT1, APOA1, CASP3, CCL5, CD14, CD36, CD9, CR2, CSF1, CXCL12, EGF, EGFR, FGFR1, HPSE, HSPB1, IGF1, IGHM, IL18, KIT, MMP14, MMP9, MSTN, PIGR, PON1, PROC, SAA1, SFTPD, SOD1, SRC, VCAM1
Neovascularization	4.9E-12	APOE, CD44, FGFR2, IGF1, MMP14, MMP2, MMP9, TIMP2, VCAM1	6.56E-12	APOE, CD44, FGFR2, IGF1, MMP14, MMP2, MMP9, TIMP2, VCAM1	3.32E-15	AIMP1, APOE, CD44, FGFR1, FGFR2, IGF1, MMP14, MMP2, MMP9, TIMP2, VCAM1	5.13E-20	AKT1, CSF1, CXCL12, EFNB2, EGF, FGFR1, HPSE, IGF1, IGF2, IL18, KIT, MMP14, MMP9, NRP1, SOD1, SRC, VCAM1
Metabolism of ROS	N/A	N/A	N/A	N/A	N/A	N/A	4.72E-19	ACE, AKT1, APOA1, APOA4, CASP3, CCL5, CD14, CD36, CSF1, CXCL12, EGF, EGFR, FADD, FAS, GPX3, GSTP1, HSPB1, IGF1, IL18, LCAT, MET, MMP14, MSTN, PRDX1, RETN, RTN4, SFTPD, SOD1, SRC, VCAM1

Diseases & Functions (HR)	Original		A1		A2		A3	
	p	Biomarker name*	*p*	Biomarker name*	*p*	Biomarker name*	*p*	Biomarker name*
**Immune mediated inflammatory disease**	1.03E-12	APOE, CD44, CSF1R, FGFR2, IGF1, IGKC, JAG1, L1CAM, MMP14, MMP2, MMP9, PRDX1, PRDX6, PTGDS, PTPRC, SELL, SFTPD, VCAM1	7.76E-15	APOE, CD44, CSF1R, FGFR2, IGF1, IGHM, IGKC, JAG1, L1CAM, MMP14, MMP2, MMP9, OSMR, PRDX1, PRDX6, PTGDS, PTPRC, SELL, SFTPD, VCAM1	1.63E-14	ANXA1, APOE, CD44, CSF1R, FGFR1, FGFR2, IGF1, IGHM, IGKC, JAG1, L1CAM, MMP14, MMP2, MMP9, PRDX1, PRDX6, PTGDS, SELL, SFTPD, VCAM1	1.97E-29	A1BG, ACE, AKT1, ALDOA, APOA1, CASP3, CCL5, CD36, CD9, COMP, CR2, CSF1, CXCL12, FADD, FAS, FGFR1, GFAP, GSTO1, HPSE, IGF1, IGF2, IGHM, IL18, KIT, KLK6, L1CAM, MBL2, MMP14, MMP9, NRP1, OSMR, PIGR, PON1, PRDX1, PROC, PTGDS, PTPRC, SAA1, SFTPD, SRC, TPM3, VCAM1, ZYX
**Inflammation of organ**	2.95E-14	APOA2, APOE, CD44, CSF1R, FGFR2, GPNMB, GPX1, IGF1, IGKC, JAG1, LDLR, MMP14, MMP2, MMP9, PRDX1, PRDX6, PROS1, PTGDS, PTPRC, SELL, SFTPD, VCAM1	4.59E-15	APOA2, APOE, CD44, CSF1R, FGFR2, GPNMB, GPX1, IGF1, IGHM, IGKC, JAG1, LDLR, MMP14, MMP2, MMP9, OSMR, PRDX1, PRDX6, PTGDS, PTPRC, SELL, SFTPD, VCAM1	1.66E-13	ANXA1, APOA2, APOE, CD44, CSF1R, FGFR2, GPNMB, GPX1, IGF1, IGHM, IGKC, JAG1, LDLR, MMP14, MMP2, MMP9, PRDX1, PRDX6, PTGDS, SELL, SFTPD, VCAM1	8.68E-26	ACE, ADK, AKT1, ALDOA, APOA1, APOA4, APOB, CASP3, CCL5, CD14, CD36, CR2, CSF1, CXCL12, EGF, EGFR, FADD, FAS, GFAP, GSTO1, GSTP1, HDAC6, HPSE, IGF1, IGF2, IGHM, IL18, KIT, KLK6, MMP14, MMP9, OSMR, PDE5A, PIGR, PON1, PRDX1, PROC, PTGDS, PTPRC, RET, SFTPD, SOD1, SRC, TPM3, VCAM1, ZYX
**Rheumatoid arthritis**	9.38E-09	APOE, CD44, IGF1, IGKC, L1CAM, MMP14, MMP2, MMP9, PRDX1, PTPRC, VCAM1	9.19E-10	APOE, CD44, IGF1, IGHM, IGKC, L1CAM, MMP14, MMP2, MMP9, PRDX1, PTPRC, VCAM1	1.34E-09	ANXA1, APOE, CD44, IGF1, IGHM, IGKC, L1CAM, MMP14, MMP2, MMP9, PRDX1, VCAM1	3.31E-19	A1BG, ALDOA, APOA1, CASP3, CCL5, CD36, COMP, CR2, CSF1, CXCL12, GFAP, IGF1, IGHM, IL18, KIT, L1CAM, MBL2, MMP14, MMP9, NRP1, PON1, PRDX1, PTPRC, SAA1, SRC, VCAM1
**Systemic autoimmune syndrome**	1.14E-08	APOE, CD44, GPNMB, IGF1, IGKC, JAG1, L1CAM, MMP14, MMP2, MMP9, PRDX1, PTPRC, SELL, VCAM1	1.74E-09	APOE, CD44, GPNMB, IGF1, IGHM, IGKC, JAG1, L1CAM, MMP14, MMP2, MMP9, PRDX1, PTPRC, SELL, VCAM1	2.8E-09	ANXA1, APOE, CD44, GPNMB, IGF1, IGHM, IGKC, JAG1, L1CAM, MMP14, MMP2, MMP9, PRDX1, SELL, VCAM1	3.58E-22	A1BG, ACE, AKT1, ALDOA, APOA1, CASP3, CCL5, CD14, CD36, CD9, COMP, CR2, CSF1, CXCL12, FADD, FAS, GFAP, HPSE, IGF1, IGHM, IL18, KIT, L1CAM, MBL2, MMP14, MMP9, NRP1, PON1, PRDX1, PROC, PTPRC, RET, RETN, SAA1, SRC, VCAM1
**Chronic inflammatory disorder**	5.21E-11	APOA2, APOE, CD44, FGFR2, GPNMB, IGF1, IGKC, L1CAM, MMP14, MMP2, MMP9, PRDX1, PTGDS, PTPRC, SELL, VCAM1	6.77E-12	APOA2, APOE, CD44, FGFR2, GPNMB, IGF1, IGHM, IGKC, L1CAM, MMP14, MMP2, MMP9, PRDX1, PTGDS, PTPRC, SELL, VCAM1	1.21E-11	ANXA1, APOA2, APOE, CD44, FGFR2, GPNMB, IGF1, IGHM, IGKC, L1CAM, MMP14, MMP2, MMP9, PRDX1, PTGDS, SELL, VCAM1	4.17E-26	A1BG, ACE, ADK, AKT1, ALDOA, APOA1, CASP3, CCL5, CD36, COMP, CR2, CSF1, CXCL12, EGF, EGFR, FAS, GFAP, HPSE, IGF1, IGHM, IL18, KIT, L1CAM, MASP2, MBL2, MMP14, MMP9, NRP1, PDE5A, PIGR, PON1, PRDX1, PROC, PTGDS, PTPRC, RET, SAA1, SRC, VCAM1

Footnote: Shown *p* values correspond to averaged values provided by *IPA* for the consolidated biomarker candidates predicted in the context of each disease and function in the proteomics and metabolomics datasets independently since *IPA* cannot currently perform biomarker predictions in integrated multi-omics datasets.

* Biomarker names correspond to the associated gene names.

Table S4B: Lists of consolidated candidate biomarkers predicted by *IPA* in the context of the shown Diseases&Functions in the proteomics and metabolomics datasets for the new-onset (NO) subject group.

Diseases & Functions (NO)	Original		A1		A2		A3	
	p	Biomarker name*	*p*	Biomarker name*	*p*	Biomarker name*	*p*	Biomarker name*
**Diabetes mellitus**	2.55E-32	ACE, ANXA1, APOA2, APOC1, APOC2, APOC3, APOE, BMP1, CD44, CD7, CETP, CR2, CXCL12, DDR1, EGFR, FABP4, FGFR1, GPNMB, GREM1, ICAM1, IGF1, IGF2, IGFBP2, IGHM, IL18, JAG1, L1CAM, LDLR, LEP, LEPR, MASP2, MEP1B, MMP14, MMP2, MMP9, NTRK2, PCSK9, PTGDS, PTPRC, RETN, SELL, SERPINE1, SFTPD, SOD1, VCAM1	4.07E-30	ACE, ANXA1, APOA2, APOC1, APOC2, APOC3, APOE, BMP1, CD44, CD7, CETP, CR2, CXCL12, DDR1, EGFR, FABP4, FGFR1, GPNMB, GREM1, ICAM1, IGF2, IGFBP2, IL18, JAG1, L1CAM, LDLR, LEP, LEPR, MASP2, MEP1B, MMP14, MMP2, MMP9, NTRK2, PCSK9, PTGDS, PTPRC, RETN, SELL, SERPINE1, SFTPD, SOD1, VCAM1	5.02E-31	ACE, ANXA1, ANXA2, APOA2, APOC1, APOC2, APOC3, APOE, BMP1, CD44, CETP, CR2, CXCL12, DDR1, EGFR, FABP4, FGFR1, GPNMB, GREM1, ICAM1, IGF1, IGF2, IGFBP2, IGHM, IL18, JAG1, L1CAM, LDLR, LEP, LEPR, MASP2, MEP1B, MMP14, MMP2, MMP9, NTRK2, PCSK9, PTGDS, PTPRC, SELL, SERPINE1, SFTPD, SOD1, VCAM1	1.31E-24	ACE, AKT1, AMBP, ANXA5, APOA4, APOB, APOC1, BMP1, CALR, CCL5, CD36, CD44, CRP, CXCL12, EGFR, FABP4, FADD, FAS, FGFR1, GFAP, GREM1, GSTO1, GSTP1, HPSE, HSPB1, HUWE1, IGF1, IGFBP2, IGHM, L1CAM, LDLR, LEPR, MASP2, MIF, MMP2, MMP9, PCSK9, PON1, PTPRC, SERPINA3, VCAM1
**Chemotaxis**	6.83E-26	ANXA1, APOE, CD44, CD9, CSF1R, CXCL12, EFNB2, EGF, EGFR, GREM1, ICAM1, IGF1, IGF2, IL18, KIT, L1CAM, LDLR, LEP, MET, MMP14, MMP2, MMP9, NRP1, PTPRC, SELL, SERPINE1, SFTPD, SLPI ,VCAM1	9.7E-25	ANXA1, APOE, CD44, CD9, CSF1R, CXCL12, EFNB2, EGF, EGFR, GREM1, ICAM1, IGF2, IL18, KIT, L1CAM, LDLR, LEP, MET, MMP14, MMP2, MMP9, NRP1, PTPRC, SELL, SERPINE1, SFTPD, SLPI, VCAM1	2.86E-27	ANXA1, ANXA2, APOE, CD44, CD9, CSF1, CSF1R, CXCL12, EFNB2, EGF, EGFR, GREM1, ICAM1, IGF1, IGF2, IL18, L1CAM, LDLR, LEP, MET, MMP14, MMP2, MMP9, NRP1, PTPRC, SELL, SERPINE1, SFTPD, SLPI, VCAM1	4.1.25E-28	AKT1, CALR, CCL5, CD36, CD44, CD9, CSF1, CSF1R, CXCL12, EGFR, FADD, FAS, GREM1, HDAC6, HPSE, HSPB1, IGF1, L1CAM, LBP, LDLR, MET, MIF, MMP2, MMP9, NTRK3, PLEC, PTPRC, RTN4, SAA1, SERPINA3, SLPI, VCAM1
**Cell movement**	9.07E-39	ANXA1, APOC1, APOE, CD14, CD44, CD9, CR2, CSF1R, CUL1, CXCL12, DDR1, EFNB2, EGF, EGFR, FABP4, FGFR1, FGFR2, FSCN1, GPNMB, GREM1, ICAM1, IGF1, IGF2, IGFBP2, IGHM, IL18, JAG1, KIT, L1CAM, LCAT, LDLR, LEP, LEPR, MASP2, MEP1B, MET, MMP14, MMP2, MMP9, NCAM1, NRP1, NTRK2, OSMR, PCSK9, PRDX1, PROC, PROS1, PTGDS, PTPRC, PTPRG, RET, RETN, SELL, SERPINE1, SFTPD, SLPI, SOD1, STAB2, TIMP2, VCAM1	1.76E-39	AIMP1, ANXA1, APOC1, APOE, CD14, CD44, CD9, CR2, CSF1R, CUL1, CXCL12, DDR1, EFNB2, EGF, EGFR, FABP4, FGFR1, FGFR2, FSCN1, GPNMB, GREM1, ICAM1, IGF2, IGFBP2, IL18, JAG1, KIT, L1CAM, LCAT, LDLR, LEP, LEPR, LYVE1, MASP2, MEP1B, MET, MMP14, MMP2, MMP9, NCAM1, NRP1, NTRK2, OSMR, PCSK9, PRDX1, PROC, PROS1, PTGDS, PTPRC, PTPRG, RET, RETN, SELL, SERPINE1, SFTPD, SLPI, SOD1, STAB2, TIMP2, VCAM1	2.98E-40	AIMP1, ANXA1, ANXA2, APOC1, APOE, CD14, CD44, CD9, CR2, CSF1, CSF1R, CUL1, CXCL12, DDR1, EFNB2, EGF, EGFR, FABP4, FGFR1, FGFR2, FSCN1, GPNMB, GREM1, ICAM1, IGF1, IGF2, IGFBP2, IGHM, IL18, JAG1, L1CAM, LCAT, LDLR, LEP, LEPR, MASP2, MEP1B, MET, MMP14, MMP2, MMP9, NCAM1, NRP1, NTRK2, OSMR, PCSK9, PRDX1, PROC, PROS1, PTGDS, PTPRC, PTPRG, RET, SELL, SERPINE1, SFTPD, SLPI, SOD1, STAB2, TIMP2, VCAM1	3.20E-37	AKT1, ALDOA, APOB, APOC1, APOH, CALR, CCL5, CD14, CD36, CD44, CD9, COMP, CRP, CSF1, CSF1R, CXCL12, EGFR, FABP4, FADD, FAS, FCN1, FGFR1, FGFR2, FSCN1, GFAP, GREM1, HDAC6, HPSE, HSPB1, HUWE1, IGF1, IGFBP2, IGHM, L1CAM, LAMA3, LBP, LCAT, LDHA, LDLR, LEPR, LYVE1, MASP2, MET, MIF, MMP2, MMP9, NCAM1, NTRK3, OSMR, PCSK9, PDIA3, PLEC, PRDX6, PTPRC, RET, RTN4, SAA1, SERPINA3, SLPI, STAB2, TPM2, TPM3, VCAM1

Diseases & Functions (NO)	Original		A1		A2		A3	
	p	Biomarker name*	*p*	Biomarker name*	*p*	Biomarker name*	*p*	Biomarker name*
Migration of cells	1.78E-38	ANXA1, APOC1, APOE, CD14, CD44, CD9, CR2, CSF1R, CUL1, CXCL12, DDR1, EFNB2, EGF, EGFR, FABP4, FGFR1, FGFR2, FSCN1, GPNMB, GREM1, ICAM1, IGF1, IGF2, IGFBP2, IL18, JAG1, KIT, L1CAM, LCAT, LDLR, LEP, LEPR, MASP2, MEP1B, MET, MMP14, MMP2, MMP9, NCAM1, NRP1, NTRK2, OSMR, PCSK9, PRDX1, PROC, PROS1, PTGDS, PTPRC, RET, RETN, SELL, SERPINE1, SFTPD, SLPI, SOD1, STAB2, TIMP2, VCAM1	1.29E-40	AIMP1, ANXA1, APOC1, APOE, CD14, CD44, CD9, CR2, CSF1R, CUL1, CXCL12, DDR1, EFNB2, EGF, EGFR, FABP4, FGFR1, FGFR2, FSCN1, GPNMB, GREM1, ICAM1, IGF2, IGFBP2, IL18, JAG1, KIT, L1CAM, LCAT, LDLR, LEP, LEPR, LYVE1, MASP2, MEP1B, MET, MMP14, MMP2, MMP9, NCAM1, NRP1, NTRK2, OSMR, PCSK9, PRDX1, PROC, PROS1, PTGDS, PTPRC, RET, RETN, SELL, SERPINE1, SFTPD, SLPI, SOD1, STAB2, TIMP2, VCAM1	6.25E-40	AIMP1, ANXA1, ANXA2, APOC1, APOE, CD14, CD44, CD9, CR2, CSF1, CSF1R, CUL1, CXCL12, DDR1, EFNB2, EGF, EGFR, FABP4, FGFR1, FGFR2, FSCN1, GPNMB, GREM1, ICAM1, IGF1, IGF2, IGFBP2, IL18, JAG1, L1CAM, LCAT, LDLR, LEP, LEPR, MASP2, MEP1B, MET, MMP14, MMP2, MMP9, NCAM1, NRP1, NTRK2, OSMR, PCSK9, PRDX1, PROC, PROS1, PTGDS, PTPRC, RET, SELL, SERPINE1, SFTPD, SLPI, SOD1, STAB2, TIMP2, VCAM1	9.64E-35	AKT1, ALDOA, APOB, APOC1, APOH, CALR, CCL5, CD14, CD36, CD44, CD9, COMP, CRP, CSF1, CSF1R, CXCL12, EGFR, FABP4, FAS, FCN1, FGFR1, FGFR2, FSCN1, GFAP, GREM1, HDAC6, HPSE, HSPB1, HUWE1, IGF1, IGFBP2, L1CAM, LAMA3, LBP, LCAT, LDHA, LDLR, LEPR, LYVE1, MASP2, MET, MIF, MMP2, MMP9, NCAM1, NTRK3, OSMR, PCSK9, PLEC, PTPRC, RET, RTN4, SAA1, SERPINA3, SLPI, STAB2, TPM2, TPM3, VCAM1
Cellular infiltration	1.32E-22	ANXA1, APOE, CD14, CD44, CSF1R, CXCL12, EGFR, FABP4, ICAM1, IGHM, IL18, LCAT, LDLR, LEP, MASP2, MET, MMP14, MMP2, MMP9, NRP1, PCSK9, PRDX1, PROC, PTGDS, RETN, SELL, SERPINE1, VCAM1	1.53E-21	ANXA1, APOE, CD14, CD44, CSF1R, CXCL12, EGFR, FABP4, ICAM1, IL18, LCAT, LDLR, LEP, MASP2, MET, MMP14, MMP2, MMP9, NRP1, PCSK9, PRDX1, PROC, PTGDS, RETN, SELL, SERPINE1, VCAM1	6.94E-24	ANXA1, ANXA2, APOE, CD14, CD44, CSF1, CSF1R, CXCL12, EGFR, FABP4, ICAM1, IGHM, IL18, LCAT, LDLR, LEP, MASP2, MET, MMP14, MMP2, MMP9, NRP1, PCSK9, PRDX1, PROC, PTGDS, SELL, SERPINE1, VCAM1	2.2E-23	AKT1, ALDOA, CCL5, CD14, CD36, CD44, CSF1, CSF1R, CXCL12, EGFR, FABP4, FAS, FCN1, GFAP, HSPB1, IGHM, LBP, LCAT, LDLR, MASP2, MET, MIF, MMP2, MMP9, PCSK9, PDIA3, PLEC, PRDX6, SAA1, VCAM1
Accumulation of cells	1.15E-23	ANXA1, APOC3, APOE, CD44, CXCL12, DDR1, EGF, EGFR, FGFR1, FGFR2, ICAM1, IGF1, IL18, KIT, LDLR, MMP9, OSMR, PTGDS, RET, SELL, SLPI, SOD1, TIMP2, VCAM1	N/A	N/A	4.13E-25	AIMP1, ANXA1, APOC3, APOE, CD44, CSF1, CXCL12, DDR1, EGF, EGFR, FGFR1, FGFR2, ICAM1, IGF1, IL18, LDLR, MMP9, OSMR, PTGDS, RET, SELL, SLPI, SOD1, TIMP2, VCAM1	N/A	N/A
Quantity of lymphocytes	8.11E-25	ANXA1, APOE, CD44, CD7, CR2, CSF1R, CXCL12, DDR1, EFNB2, FGFR1, FGFR2, ICAM1, IGF1, IGF2, IGHM, IGKC, IL18, JAG1, KIT, LDLR, LEP, LEPR, MMP9, MSH2, PRDX1, PROC, PTPRC, SELL, SFTPD, SOD1, VCAM1	1.63E-22	ANXA1, APOE, CD44, CD7, CR2, CSF1R, CXCL12, DDR1, EFNB2, FGFR1, FGFR2, ICAM1, IGF2, IGKC, IL18, JAG1, KIT, LDLR, LEP, LEPR, MMP9, MSH2, PRDX1, PROC, PTPRC, SELL, SFTPD, SOD1, VCAM1	1.5E-23	ANXA1, APOE, CD44, CR2, CSF1, CSF1R, CXCL12, DDR1, EFNB2, FGFR1, FGFR2, ICAM1, IGF1, IGF2, IGHM, IGKC, IL18, JAG1, LDLR, LEP, LEPR, MMP9, MSH2, PRDX1, PROC, PTPRC, SELL, SFTPD, SOD1, VCAM1	2.92E-17	AKT1, AP3B1, CCL5, CD36, CD44, CRP, CSF1, CSF1R, CXCL12, FADD, FAS, FGFR1, FGFR2, GSTP1, IGF1, IGHM, LAMA3, LDLR, LEPR, MIF, MMP9, MSH2, PAWR, PTPRC, SAA1, VCAM1
Quantity of myeloid cells	2.37E-22	ANXA1, APOE, CD9, CR2, CSF1R, CXCL12, DDR1, GREM1, ICAM1, IGF1, IL18, KIT, LCAT, LDLR, LEP, LEPR, MMP9, PRDX1, PROC, PROS1, SELL, SERPINE1, SFTPD, SLPI, SOD1, TIMP2, VCAM1	2.9E-21	ANXA1, APOE, CD9, CR2, CSF1R, CXCL12, DDR1, GREM1, ICAM1, IL18, KIT, LCAT, LDLR, LEP, LEPR, MMP9, PRDX1, PROC, PROS1, SELL, SERPINE1, SFTPD, SLPI, SOD1, TIMP2, VCAM1	2.37E-22	ANXA1, APOE, CD9, CR2, CSF1, CSF1R, CXCL12, DDR1, GREM1, ICAM1, IGF1, IL18, LCAT, LDLR, LEP, LEPR, MMP9, PRDX1, PROC, PROS1, SELL, SERPINE1, SFTPD, SLPI, SOD1, TIMP2, VCAM1	N/A	N/A

Diseases & Functions (NO)	Original		A1		A2		A3	
	p	Biomarker name*	*p*	Biomarker name*	*p*	Biomarker name*	*p*	Biomarker name*
Quantity of T lymphocytes	7.97E-21	ANXA1, APOE, CD44, CD7, DDR1, EFNB2, FGFR1, FGFR2, ICAM1, IGF1, IGF2, IGHM, IL18, JAG1, KIT, LDLR, LEP, LEPR, MMP9, PRDX1, PROC, PTPRC, SELL, SFTPD, VCAM1	1.73E-18	ANXA1, APOE, CD44, CD7, DDR1, EFNB2, FGFR1, FGFR2, ICAM1, IGF2, IL18, JAG1, KIT, LDLR, LEP, LEPR, MMP9, PRDX1, PROC, PTPRC, SELL, SFTPD, VCAM1	1.45E-19	ANXA1, APOE, CD44, CSF1, DDR1, EFNB2, FGFR1, FGFR2, ICAM1, IGF1, IGF2, IGHM, IL18, JAG1, LDLR, LEP, LEPR, MMP9, PRDX1, PROC, PTPRC, SELL, SFTPD, VCAM1	N/A	N/A
Proliferation of immune cells	2.35E-25	ANXA1, APOE, CD14, CD44, CR2, CSF1R, CUL1, CXCL12, EFNB2, FABP4, GPNMB, ICAM1, IGF1, IGF2, IGFBP2, IGHM, IGKC, IL18, JAG1, KIT, LDLR, LEP, LEPR, MET, MMP9, MSH2, PROC, PTPRC, SELL, SERPINE1, SFTPD, SLPI, VCAM1	4.14E-23	ANXA1 ,APOE, CD14, CD44, CR2, CSF1R, CUL1, CXCL12, EFNB2, FABP4, GPNMB, ICAM1, IGF2, IGFBP2, IGKC, IL18, JAG1, KIT, LDLR, LEP, LEPR, MET, MMP9, MSH2, PROC, PTPRC, SELL, SERPINE1, SFTPD, SLPI, VCAM1	2.35E-25	ANXA1, APOE, CD14, CD44, CR2, CSF1, CSF1R, CUL1, CXCL12, EFNB2, FABP4, GPNMB, ICAM1, IGF1, IGF2, IGFBP2, IGHM, IGKC, IL18, JAG1, LDLR, LEP, LEPR, MET, MMP9, MSH2, PROC, PTPRC, SELL, SERPINE1, SFTPD, SLPI, VCAM1	7.81E-19	ACLY, AKT1, APOH, CALR, CCL5, CD14, CD36, CD44, CRP, CSF1, CSF1R, CXCL12, FABP4, FADD, FAS, GSTP1, IGF1, IGFBP2, IGHM, LDLR, LEPR, MET, MIF, MMP9, MSH2, PAWR, PTPRC, SLPI, VCAM1
Proliferation of lymphocytes	9.97E-22	ANXA1, APOE, CD14, CD44, CR2, CUL1, CXCL12, EFNB2, FABP4, GPNMB, ICAM1, IGF1, IGF2, IGFBP2, IGHM, IGKC, IL18, JAG1, KIT, LEP, LEPR, MMP9, MSH2, PROC, PTPRC, SELL, SFTPD, SLPI, VCAM1	1.51E-19	ANXA1, APOE, CD14, CD44, CR2, CUL1, CXCL12, EFNB2, FABP4, GPNMB, ICAM1, IGF2, IGFBP2, IGKC, IL18, JAG1, KIT, LEP, LEPR, MMP9, MSH2, PROC, PTPRC, SELL, SFTPD, SLPI, VCAM1	1.57E-20	ANXA1, APOE, CD14, CD44, CR2, CUL1, CXCL12, EFNB2, FABP4, GPNMB, ICAM1, IGF1, IGF2, IGFBP2, IGHM, IGKC, IL18, JAG1, LEP, LEPR, MMP9, MSH2, PROC, PTPRC, SELL, SFTPD, SLPI, VCAM1	N/A	N/A
Immune response of cells	1.3E-19	ACE, ANXA1, APOA2, APOE, CD14, CD44, CSF1R, GSTT1, ICAM1, IGHM, IL18, LDLR, LEP, LEPR, MEP1B, MET, MSH2, PROC, PROS1, PTPRC, RET, SERPINE1, SFTPD, SOD1, STAB2, VCAM1	1.93E-17	ACE, ANXA1, APOA2, APOE, CD14, CD44, CSF1R, ICAM1, IL18, LDLR, LEP, LEPR, MEP1B, MET, MSH2, PROC, PROS1, PTPRC, RET, SERPINE1, SFTPD, SOD1, STAB2, VCAM1	8.07E-21	ACE, ANXA1, APOA2, APOE, CD14, CD44, CSF1, CSF1R, GSTT1, ICAM1, IGHM, IL18, LDLR, LEP, LEPR, MEP1B, MET, MSH2, PROC, PROS1, PTPRC, RET, SERPINE1, SFTPD, SOD1, STAB2, VCAM1	3.89E-18	ACE, ANXA5, CALR, CD14, CD36, CD44, CRP, CSF1, CSF1R, FADD, FAS, FCN1, GSTT1, HSPB1, IGHM, LDLR, LEPR, MET, MIF, MSH2, PON1, PTPRC, RET, SAA1, STAB2, VCAM1
Inflammatory response	9.05E-28	ACE, ANXA1, APOE, CD14, CD44, CD9, CR2, CSF1R, CUL1, CXCL12, EGF, EGFR, FABP4, FGFR1, GPX1, GREM1, ICAM1, IGF1, IGHM, IL18, KIT, LDLR, LEP, MEP1B, MMP14, MMP2, MMP9, MSH2, PROC, PROS1, SELL, SERPINE1, SFTPD, SLPI, SOD1, VCAM1	9.18E-27	ACE, AIMP1, ANXA1, APOE, CD14, CD44, CD9, CR2, CSF1R, CUL1, CXCL12, EGF, EGFR, FABP4, FGFR1, GPX1, GREM1, ICAM1, IL18, KIT, LDLR, LEP, MEP1B, MMP14 ,MMP2, MMP9, MSH2, PROC, PROS1, SELL, SERPINE1, SFTPD, SLPI, SOD1, VCAM1	2.17E-30	ACE, AIMP1, ANXA1, ANXA2, APOE, CD14, CD44, CD9, CR2, CSF1, CSF1R, CUL1, CXCL12, EGF, EGFR, FABP4, FGFR1, GPX1, GREM1, ICAM1, IGF1, IGHM, IL18, LDLR, LEP, MEP1B, MMP14, MMP2, MMP9, MSH2, PROC, PROS1, SELL, SERPINE1, SFTPD, SLPI, SOD1, VCAM1	1.71E-25	ACE, AKT1, AP3B1, APOH, CALR, CCL5, CD14, CD36, CD44, CD9, CRP, CSF1, CSF1R, CXCL12, EGFR, FABP4, FGFR1, GREM1, HPSE, HSPB1, IGF1, IGHM, LBP, LDLR, MIF, MMP2, MMP9, MSH2, PLEC, PON1, PRDX5, PRDX6, SAA1, SERPINA3, SLPI, VCAM1
Neovasculariz ation	1.14E-25	APOE, CD44, CXCL12, EFNB2, EGF, FGFR1, FGFR2, ICAM1, IGF1, IGF2, IL18, KIT, LEP, MMP14, MMP2, MMP9, NRP1, SOD1, TIMP2, VCAM1	8.27E-26	AIMP1, APOE, CD44, CXCL12, EFNB2, EGF, FGFR1, FGFR2, ICAM1, IGF2, IL18, KIT, LEP, MMP14, MMP2, MMP9, NRP1, SOD1, TIMP2, VCAM1	3.64E-29	AIMP1, ANXA2, APOE, CD44, CSF1, CXCL12, EFNB2, EGF, FGFR1, FGFR2, ICAM1, IGF1, IGF2, IL18, LEP, MMP14, MMP2, MMP9, NRP1, SOD1, TIMP2, VCAM1	N/A	N/A

Diseases & Functions (NO)	Original		A1		A2		A3	
	p	Biomarker name*	*p*	Biomarker name*	*p*	Biomarker name*	*p*	Biomarker name*
**Metabolism of ROS**	N/A	N/A	N/A	N/A	N/A	N/A	1.83E-15	ACE, AKT1, APOA4, CCL5, CD14, CD36, CD44, CRP, CSF1, CXCL12, EGFR, FADD, FAS, GPX3, GSTP1, HSPB1, IGF1, LCAT, LDHA, LEPR, MET, MIF, PRDX5, PRDX6, RTN4, SERPINA3, VCAM1
**Immune mediated inflammatory disease**	4.51E-24	ACE, ANXA1, APOE, BMP1, CD44, CD9, CR2, CSF1R, CXCL12, DDR1, FABP4, FGFR1, FGFR2, ICAM1, IGF1, IGF2, IGHM, IGKC, IL18, JAG1, KIT, L1CAM, LEP, LEPR, MMP14, MMP2, MMP9, NRP1, NTRK2, OSMR, PRDX1, PROC, PTGDS, PTPRC, SELL, SFTPD, VCAM1	4.58E-22	ACE, ANXA1, APOE, BMP1, CD44, CD9, CR2, CSF1R, CXCL12, DDR1, FABP4, FGFR1, FGFR2, ICAM1, IGF2, IGKC, IL18, JAG1, KIT, L1CAM, LEP, LEPR, MMP14, MMP2, MMP9, NRP1, NTRK2, OSMR, PRDX1, PROC, PTGDS, PTPRC, SELL, SFTPD, VCAM1	4.51E-24	ACE, ANXA1, APOE, BMP1, CD44, CD9, CR2, CSF1, CSF1R, CXCL12, DDR1, FABP4, FGFR1, FGFR2, ICAM1, IGF1, IGF2, IGHM, IGKC, IL18, JAG1, L1CAM, LEP, LEPR, MMP14, MMP2, MMP9, NRP1, NTRK2, OSMR, PRDX1, PROC, PTGDS, PTPRC, SELL, SFTPD, VCAM1	3.56E-32	A1BG, ACE, ACLY, AKT1, ALDOA, ANXA5, APOH, BMP1, CALR, CCL5, CD36, CD44, CD9, COMP, CRP, CSF1, CSF1R, CXCL12, FABP4, FADD, FAS, FCN1, FGFR1, FGFR2, GFAP, GSTO1, HPSE, IGF1, IGHM, L1CAM, LBP, LEPR, MIF, MMP2, MMP9, OSMR, PAWR, PDIA3, PON1, PRDX5, PRDX6, PTPRC, SAA1, TPM2, TPM3, VCAM1
**Inflammation of organ**	5.75E-28	ACE, ANXA1, APOA2, APOE, CD14, CD44, CD7, CR2, CSF1R, CXCL12, DDR1, EGF, EGFR, FABP4, FCN2, FGFR2, GPNMB, GPX1, ICAM1, IGF1, IGF2, IGHM, IGKC, IL18, JAG1 ,KIT, LDLR, LEP, LEPR, MMP14, MMP2, MMP9, NTRK2, OSMR, PRDX1, PROC, PROS1, PTGDS, PTPRC, RET, SELL, SERPINE1, SFTPD, SLPI, SOD1, VCAM1	5.3E-26	ACE, ANXA1, APOA2, APOE, CD14, CD44, CD7, CR2, CSF1R, CXCL12, DDR1, EGF, EGFR, FABP4, FCN2, FGFR2, GPNMB, GPX1, ICAM1, IGF2, IGKC, IL18, JAG1, KIT, LDLR, LEP, LEPR, MMP14, MMP2, MMP9, NTRK2, OSMR, PRDX1, PROC, PROS1, PTGDS, PTPRC, RET, SELL, SERPINE1, SFTPD, SLPI, SOD1, VCAM1	8.65E-27	ACE, ANXA1, APOA2, APOE, CD14, CD44, CR2, CSF1, CSF1R, CXCL12, DDR1, EGF, EGFR, FABP4, FCN2, FGFR2, GPNMB, GPX1, ICAM1, IGF1, IGF2, IGHM, IGKC, IL18, JAG1, LDLR, LEP, LEPR, MMP14, MMP2, MMP9, NTRK2, OSMR, PRDX1, PROC, PROS1, PTGDS, PTPRC, RET, SELL, SERPINE1, SFTPD, SLPI, SOD1, VCAM1	8.25E-25	ACE, AKT1, ALDOA, AMBP, ANXA5, APOA4, APOB, APOH, CALR, CCL5, CD14, CD36, CD44, CRP, CSF1, CSF1R, CXCL12, EGFR, FABP4, FADD, FAS, FCN2, FGFR2, GFAP, GSTO1, GSTP1, HDAC6, HPSE, IGF1, IGHM, LBP, LDLR, LEPR, MIF, MMP2, MMP9, OSMR, PDIA3, PON1, PRDX6, PTPRC, RET, SERPINA3, SLPI, TPM3, VCAM1
**Rheumatoid arthritis**	N/A	N/A	N/A	N/A	N/A	N/A	2.85E-22	A1BG, ACLY, ALDOA, BMP1, CALR, CCL5, CD36, CD44, COMP, CRP, CSF1, CXCL12, FABP4, FCN1, GFAP, IGF1, IGHM, L1CAM, LBP, MIF, MMP2, MMP9, PDIA3, PON1, PRDX5, PTPRC, SAA1, TPM2, VCAM1
**Systemic autoimmune syndrome**	2.01E-21	ACE, ANXA1, APOE, BMP1, CD14, CD44, CD7, CD9, CR2, CXCL12, DDR1, FABP4, GPNMB, ICAM1, IGF1, IGHM, IGKC, IL18, JAG1, KIT, L1CAM, LEP, MMP14, MMP2, MMP9, NRP1, NTRK2, PRDX1, PROC, PTPRC, RET, RETN, SELL, VCAM1	1.77E-19	ACE, ANXA1, APOE, BMP1, CD14, CD44, CD7, CD9, CR2, CXCL12, DDR1, FABP4, GPNMB, ICAM1, IGKC, IL18, JAG1, KIT, L1CAM, LEP, MMP14, MMP2, MMP9, NRP1, NTRK2, PRDX1, PROC, PTPRC, RET, RETN, SELL, VCAM1	2.98E-19	ACE, ANXA1, APOE, BMP1, CD14, CD44, CD9, CR2, CSF1, CXCL12, DDR1, FABP4, GPNMB, ICAM1, IGF1, IGHM, IGKC, IL18, JAG1, L1CAM, LEP, MMP14, MMP2, MMP9, NRP1, NTRK2, PRDX1, PROC, PTPRC, RET, SELL, VCAM1	1.49E-22	A1BG, ACE, ACLY, AKT1, ALDOA, BMP1, CALR, CCL5, CD14, CD36, CD44, CD9, COMP, CRP, CSF1, CXCL12, FABP4, FADD, FAS, FCN1, GFAP, HPSE, IGF1, IGHM, L1CAM, LBP, MIF, MMP2, MMP9, PDIA3, PON1, PRDX5, PTPRC, RET, SAA1, TPM2, VCAM1

Diseases & Functions (NO)	Original		A1		A2		A3	
	p	Biomarker name*	*p*	Biomarker name*	*p*	Biomarker name*	*p*	Biomarker name*
**Chronic inflammatory disorder**	1.33E-26	ACE, ANXA1, APOA2, APOE, BMP1, CD44, CR2, CXCL12, DDR1, EGF, EGFR, FABP4, FGFR2, GPNMB, ICAM1, IGF1, IGHM, IGKC, IL18, KIT, L1CAM, LEP, LEPR, MASP2, MMP14 ,MMP2, MMP9, NRP1, NTRK2, PRDX1, PROC, PTGDS, PTPRC, RET, SELL, SERPINE1, SLPI, VCAM1	1.76E-24	ACE, ANXA1, APOA2, APOE, BMP1, CD44, CR2, CXCL12, DDR1, EGF, EGFR, FABP4, FGFR2, GPNMB, ICAM1, IGKC, IL18, KIT, L1CAM, LEP, LEPR, MASP2, MMP14, MMP2, MMP9, NRP1, NTRK2, PRDX1, PROC, PTGDS, PTPRC, RET, SELL, SERPINE1, SLPI, VCAM1	1.33E-26	ACE, ANXA1, APOA2, APOE, BMP1, CD44, CR2, CSF1, CXCL12, DDR1, EGF, EGFR, FABP4, FGFR2, GPNMB, ICAM1, IGF1, IGHM, IGKC, IL18, L1CAM, LEP, LEPR, MASP2, MMP14, MMP2, MMP9, NRP1, NTRK2, PRDX1, PROC, PTGDS, PTPRC, RET, SELL, SERPINE1, SLPI, VCAM1	1.8E-26	A1BG, ACE, ACLY, AKT1, ALDOA, BMP1, CALR, CCL5, CD36, CD44, COMP, CRP, CSF1, CXCL12, EGFR, FABP4, FAS, FCN1, FGFR2, GFAP, HPSE, IGF1, IGHM, L1CAM, LBP, LEPR, MASP2, MIF, MMP2, MMP9, PDIA3, PON1, PRDX5, PTPRC, RET, SAA1, SERPINA3, SLPI, TPM2, VCAM1

Footnote: Shown *p* values correspond to averaged values provided by *IPA* for the consolidated biomarker candidates predicted in the context of each disease and function in the proteomics and metabolomics datasets independently since *IPA* cannot currently perform biomarker predictions in integrated multi-omics datasets.

* Biomarker names correspond to the associated gene names.
